# Making Sense of Mismatch Negativity

**DOI:** 10.3389/fpsyt.2020.00468

**Published:** 2020-06-11

**Authors:** Kaitlin Fitzgerald, Juanita Todd

**Affiliations:** School of Psychology, University of Newcastle, Callaghan, NSW, Australia

**Keywords:** mismatch negativity, MMN, predictive coding, stimulus specific adaptation, auditory

## Abstract

Evoked potentials provide valuable insight into brain processes that are integral to our ability to interact effectively and efficiently in the world. The mismatch negativity (MMN) component of the evoked potential has proven highly informative on the ways in which sensitivity to regularity contributes to perception and cognition. This review offers a compendium of research on MMN with a view to scaffolding an appreciation for its use as a tool to explore the way regularities contribute to predictions about the sensory environment over many timescales. In compiling this work, interest in MMN as an index of sensory encoding and memory are addressed, as well as attention. Perspectives on the possible underlying computational processes are reviewed as well as recent observations that invite consideration of how MMN relates to how we learn, what we learn, and why.

## Objective

In this special issue, the reader is invited to consider “sensory information processing abnormalities in Schizophrenia and related neuropsychiatric disorders”. No issue on this topic would be complete with addressing apparent anomalies in the auditory event-related potential (ERP) component known as mismatch negativity (MMN). However, the growth in papers on MMN in schizophrenia since its first observation in 1991 (1) is formidable, and furthermore, it is exceeded by growth in the various applications for, and changes in the understanding of, MMN more generally. In this paper, we provide a review of MMN from fundamental background through to controversial new applications and in doing so we endeavor to present a perspective that represents a balance between a comprehensive and comprehensible scaffold for making sense of MMN.

## Background

To perceive, interact with, and learn about our world is perhaps the most impressive of everyday feats. We access the world by little more than the cumulative activation of sensory neurons used to build a useful representation of an environment that is endlessly complex. In doing so, we are limited by the fact that our environment is richer in information than a limited and noisy sensory system could ever fully attend to, and the information itself is often imperfect. To properly understand sensation and perception therefore requires understanding both how sensation is produced in the world, and how our sensory systems could construct a meaningful representation of the world from these sensations especially when the information carried is uncertain. Bregman (2) defined the “job” of perception as “to take sensory input and to derive a useful representation of reality from it”. It is a challenging yet vital task that sensations are rapidly perceived and organized in order to guide adaptive behavior.

Studies of brain function have revealed strategies that may help simplify sensory processing by reducing the resources required for adequate perception. These strategies involve “short cuts” or heuristics, where assumptions are made which invite some possibility of error. One example is in the processing of repetition, where brain responses are observed to be smaller to a repeated stimulus compared to an equivalent novel stimulus. Predictive coding is a dominant theoretical model for this process, which sits among several alternative accounts that will be contrasted later in this review. These models recognize that our world is both ever-changing and constrained by regularity, and it is of little benefit to process a repeated stimulus as if we are encountering it for the first time on each repetition. Predictive coding in particular suggests that the brain is sensitive to the rate at which stimuli have occurred in the recent past and uses this information to actively infer the future state of the world (3, 4). That is, repeated and predicted stimuli require little effort to process, while neural resources are prioritized for processing novel events which are more likely to carry new and behaviorally relevant information. While this process is seemingly labor-intensive, the result is ultimately a more parsimonious use of neural resources which facilitates the complex task of translating sensation to perception.

This review culminates into a discussion of another possible heuristic—a *first-impression bias* in predictive coding where initial learning about the probability, transitions, and importance of a sound influences how that sound is later processed even after conditions change (5–8). This effect has been shown *via* the application of electroencephalography (EEG), a common neuroimaging technique, to study the MMN component of the ERP. MMN is well supported as an index of automatic change-detection which is elicited following any change to an established regularity in sensory stimuli, including sound, with its amplitude providing some quantification of the salience of the unexpected stimulus for processing [see (9–12) for reviews]. From a predictive coding perspective, MMN is viewed as a “prediction error signal” which can be used to study how the brain monitors environmental statistics to detect regularity and change, and generate top-down predictions which facilitate stimulus processing (3, 12, 13). This assertion was based on the notion of a system which adjusts rapidly to change in order to maximize predictive accuracy. However, the first-impression bias shows that to the contrary, the categorization of a stimulus when first encountered can be perseverative [e.g., (7, 8, 14, 15)].

## The Case for Auditory Processing Heuristics

Auditory signals are often immediately informative for behavior (consider the urgency with which we respond to fire alarms, car horns, and cooking timers), yet are complex sensory signals to process. Auditory input can be endlessly layered; a single signal consisting of the summed output of every sound-producing object present in the environment at any given time (2). Representations of the sound environment and its constituent objects must be derived solely from temporal changes in pressure in this single composite signal imposed upon the ear. The transient nature of auditory input also leaves a limited time window for this complex process to occur. Auditory processing therefore presents a particular challenge to translate a complex signal under significant time pressure and respond with adaptive behavior.

A solution to the problem of complex processing in any limited system is the implementation of heuristics or short-cuts that serve to reduce and expedite processing. In an adaptive organism, these mechanisms should reflect an optimal accuracy-effort trade-off, where the chance of a negligible degree of error is accepted in exchange for an overall reduction in processing effort. Fortunately, for perception, we can often successfully apply the assumption that the macroscopic world has order. Therefore, a useful basis for heuristic processing in perception is patterns or regularities in the environment. There are many examples of the brain's use of these patterns to manage complexity and uncertainty. For example, in auditory scene analysis, the auditory system parses the single chaotic auditory signal into meaningful representations of discrete auditory objects on the assumption of ecologically valid regularities (2). Sounds are grouped on the basis of shared characteristics that increase the probability that they originate from the same source, such as consistent timber, continuation of a pattern or feature (e.g., step-wise ascending frequency), or termination at the same point in time. Heuristics also provide a means to infer sensory information that is lacking.

Bayesian perspectives provide a formal account of how regularities in the world are exploited to support perception. These models assert that complexity and uncertainty is optimally resolved through the use of probability statistics (16). When sensory data is missing or unreliable, it is inferred based on the relative probabilities of all possible states (e.g., possible distance from an object) and the likelihood they would produce the current data [e.g., that an auditory signal at a given distance would produce a sound of the current intensity (17)]. In contrast to frequentist statistics where conditional probability is calculated from many trials, a Bayesian approach represents probability as the likelihood an event will occur based on prior experience as well as previously held beliefs or information. Bayes' theorem specifies that the conditional probability of an event A given event B can be estimated as P(A|B) = P(B|A) P(A)/P(B), where P(A) represents a prior probability based on previously held beliefs about A, and P(B) represents some new data or observation related to event A. New information about event B causes P(B) to be updated, leading to a new calculation of posterior probability P(A|B), or an updating of one's prior beliefs about P(A) when new evidence P(B) is generated. Optimal perception is achieved when these estimates are then given appropriate weighting based on their uncertainty (16).

Importantly, the use of probability naturally entails that perception is not infallible but reflects an optimal effort/accuracy trade-off where some likelihood of error is accepted in exchange for the conservation of neural resources. Sensory illusions provide examples of the type of negligible errors that can occur when this shallower processing approach is adopted. To maintain an optimal level of accuracy, it is vital that the system can detect and differentiate potentially meaningful errors in order to adjust its estimates of the world accordingly. MMN, as a distinct component of the ERP that is elicited only by a detected change to an established regularity in the environment, has been isolated as a distinct neural marker of such error and change detection (3, 12, 13).

## Introduction to the MMN

### General Characteristics

The auditory MMN is an evoked response that appears in neurophysiological recordings as a brief negative deflection in amplitude following a sound that deviates from some established repetition or consistency in the recent past (18). In a laboratory setting, MMN is typically studied using an oddball paradigm, where it is observed following each occurrence of a low-probability “deviant” sound irregularly interspersed among a series of highly repetitive “standard” sounds from which it differs on some dimension (18–20). This additional negative component is most easily observed in a difference waveform produced from the subtraction of the response to the standard from that to the deviant. Onset is observed as early as 50 ms with a peak 100- to 250-ms post-stimulus, though latency and amplitude does vary with the specific characteristics of the sound sequence [(11, 21); see (22, 23) for reviews]. In ERP scalp recordings with the nose as reference, MMN is maximal at fronto-central electrode sites, often with a right-hemisphere preponderance, with a polarity inversion of this component at sites located at and around the mastoid bone (24). **Table 1** presents a summary of many of the variables observed to impact MMN as reviewed below.

**Table 1:** A number of variables observed to affect MMN amplitude*.

Variable	Example reference
Time point of deviation	Picton et al. (11)
Discrimination difficulty	Sams et al. (21)
Number of regularities violated	Schröger and Wolff (25)
Strength of memory trace	Baldeweg et al. (26)
The way sounds are grouped	Cowan et al. (27)
Backward masking	Winkler and Näätänen (28)
Variability in the repetition	Winkler et al. (29).
Local probability of the deviant	Csépe et al. (30)
Period of stable regularity	Todd et al. (8)
Level of attention	Woldorff et al. (31)
Familiarity or salience	Korzyukov et al. (32)
Order of sound regularities	Todd et al. (8)
Brain lesions (e.g., frontal cortex)	Alain et al. (33)
Temporary disruption to frontal cortex	Weigl et al. (34)
Knowledge of sound structure	Sussman et al. (35).
Nature of experimental control for SSA	Jacobsen et al. (36)
Volatility in initial sequence segments	Todd et al. (37)
Clinical conditions and aging	Näätänen et al. (38). Review.

*More extensive examples provided in text.

### Discriminability

Early studies confirmed the separability of MMN from the highly similar N1 and N2b negative components on which it is often superimposed (18, 21, 39). The N1 is an exogenous response to the change in energy posed by a stimulus and is therefore observed to both standard and deviant tones (40). The N1 shows directional modulation, decreasing in amplitude with decreasing intensity of the stimulus whereas MMN reflects only the absolute value of a difference between standard and deviant stimuli [(19); see (11) for a review]. The N2b follows MMN [also referred to as the auditory N2a; e.g., (39, 41)] as a latter subcomponent that occurs only when the deviant stimulus is consciously and voluntarily processed, whereas MMN persists in the absence of conscious attention (20, 42, 43). Anatomically, MMN is unique in that it is more anterior than both N1 and N2b with modality-specific variations in topography [see (44) for a review] and is likely produced by distinct cortical sources (45–47). The reversal of polarity at mastoid sites in nose-referenced recordings is also unique to MMN and presents a useful way to isolate a measure of “pure” MMN from this overlapping N2b subcomponent (21, 48, 49).

Functionally, MMN is defined by two key characteristics: that it is context-dependent and does not rely on conscious attention to the stimulus. Whereas both N1 and N2b can be elicited by a deviant stimulus alone, MMN occurs only when the sound is interspersed among a series of repetitive standards (27, 45). Where N2b is only elicited when deviant stimuli are consciously attended and N1 is highly prone to modulation by attention, MMN will be observed to deviations in both attended and unattended stimulus streams and is far less permeable to attention effects [(21, 39); but see also (50)]. MMN is also independent of the later P3 component which reflects stimulus significance and attentional capture (51, 52). Cleverly designed control paradigms have ruled out the possibility that MMN could be an artefact of effects on these other exogenous components, cementing MMN as a distinct component that uniquely reflects stimulus discrimination and change detection processes [see (53) for a review]. The MMN has since become the most widely utilized method of studying same.

### MMN and Sensory Memory

MMN generation is assumed to rest on the comparison of the incoming deviant stimulus to a stored neural representation of the standard, and can thereby provide a putative index of sensory memory formation and decay. MMN will only be elicited to a deviant sound presented in a stream of repetitive standards when it is sufficiently rare [probability of 0.30 or below (54)]. This sensitivity betrays two features of this change detection mechanism: (1) the ability to detect the actual physical difference in sensations, and (2) the extraction of patterns in sound and their relative probabilities. Both processes are dependent on the formation and short-term maintenance of a memory trace for the standard and deviant sound, rendering MMN a useful probe for the formation of sensory memory representations and their discrimination [(19); see (55) for a review]. In this review, the term sensory memory is used to refer to the brief retention of information about a sound that has just occurred, and we assume it adheres to estimated limits associated with passive memory decay [e.g., (56)]. Meanwhile, the term memory trace refers more broadly to any activated (or reactivated) state which includes, but is not restricted to, sensory memory. A predictive model at minimum is supposed to entail the additional property of being a memory trace associated with probability estimates regarding the likely “next state” (i.e., transition probabilities).

#### Encoding

MMN will be elicited following a deviation in any sound characteristic [e.g., frequency, intensity, duration, location; see (11, 19) for reviews]. Deviations may be characterized by simple departures from a single static feature of a repeated sound, or more complex regularities formed across multiple features of single tones or repeated tone pairs or groupings [e.g., changes in a repeated 5-tone serial sequence with a short stimulus-onset asynchrony (57), or an unexpected repetition within a series of two consistently alternating tones (58)]. The change may also be built into the experimental design of the sequence, such as changes in the interstimulus interval (ISI) in a stream of physically identical tone bursts (59). MMN also shows sensitivity to “abstract” deviations such as a change in the relative interval or direction of differences between adjacent tones [e.g., an occasional descending-frequency tone pair among a series of ascending-frequency tone pairs where no absolute characteristics of the tones are shared to form a physical or “first-order” standard (60); see (61) for a review] or where there is an unexpected stimulus omissions (62–64). Therefore, it is important to note that the terms “standard” and “deviant” refer not to individual tones necessarily, but rather the neural representations of a regularity and a violating event which can vary in complexity (12).

#### Discrimination

MMN latency is also highly variable and is considered to index the nature and difficulty of the standard-deviant comparison process as it is assumed that MMN will only be elicited after some “decision point” where an uncommon change is realized (65). This decision point may be impacted by the actual point of difference between the stimuli [e.g., will occur later for a longer-duration deviant than a shorter-duration deviant (11)] as well as discrimination difficulty. MMN latency is reduced where the two tones are more clearly distinct [(21, 51, 66); see (10) for a review] and will extend to as long as 200–300 ms in the case of barely discriminable differences (67).

MMN amplitude is also taken to reflect some quantification of discrimination difficulty (10). Broadly speaking, measured MMN can increase with two factors likely related to the clarity or certainty of a change: (1) the degree of physical difference between the repetitive and deviant stimulus, and (2) some quantification of the “strength” with which the regularity is encoded [the exact interpretation of this variable varies among models of MMN, as will later be discussed (12)]. MMN is larger when the difference between the standard and deviant is more marked, whether this is due to a greater degree of physical difference between the tones (19, 68) or concurrent deviation on multiple stimulus dimensions (10, 25). MMN amplitude appears to reflect the strength of the memory trace for the standard, increasing with the number of consecutive standards (26, 27, 69, 70), reduced ISI between sounds (71), and is reduced by backward masking the standard (28). Meanwhile, modulations associated with the degree of variability in sound have led to the assertion that MMN may additionally reflect some estimate of certainty or accuracy of this memory trace. MMN amplitude will increase with decreased variability in the characteristics of the standard (29), smaller local probability of the deviant (19, 30), and the overall period of time that a regularity has been stable [e.g., (8); but see later discussion of this study].

### MMN and Attention

Another important feature of MMN is that it can be observed without conscious attention to the sound stream, suggesting that sophisticated sensory discrimination processes are initiated at the pre-attentive level (72–74). Observations of MMN have been made across passive listening conditions (21, 75), states of reduced consciousness such as coma and sleep (76–78), and in the absence of behavioral discrimination ability (31, 79). These observations have led to the conclusion that MMN is pre-attentive and reflects some “primitive intelligence” within the auditory cortex (18, 80, 81).

However, modulations of MMN amplitude with attention challenge the extent to which MMN can be considered truly pre-attentive. While a number of studies have displayed no difference in amplitude across ignored and attended sound streams (73, 74, 82, 83), an equally strong body of research has shown systematic increases in MMN amplitude with the level of conscious attention to a deviant (84–87). In an attempt to reconcile these findings, it has been suggested that attention effects reflect biased encoding of the memory trace for the standard, but deviant detection itself remains impermeable to attention (35, 88). MMN may therefore be best conceptualized as an index of sensory memory representations which is not dependent on attention, but can be manipulated by the effect of attention on how sensory memory representations are formed.

Automatic deviance detection is conversely thought to have implications for attention by serving as an information filter—a bottom-up signal of new information that can redirect attention toward the deviant sound. Source localization has consistently identified a frontal contribution to MMN generation which is thought to be responsible for this proposed attention switch [(24, 89, 90); c.f. (91)]. Frontal cortices have a specialized role in selective attention and orienting (92, 93) and typically show the same right-hemisphere preponderance which has been observed for MMN at frontal electrode sites [(91, 94, 95); however see (96) for discussion of left-lateralized MMN to speech and language deviants]. In accordance with this idea, MMN is regularly followed by the P3a component which is considered a neural indicator of involuntary attention capture with origins in frontal cortex (97–100). The three-stage model of involuntary attention (23, 101) assumes that MMN is responsible for initiating a series of upstream processes related to further evaluation of the deviant event (102). Specifically, this involves an involuntary direction of attention toward and subsequent evaluation of this change indexed by P3a (99), and the re-direction of attention back to the task at hand indexed by the reorienting negativity (25).

An important aspect of involuntary attention is the ability to appropriately filter relevant change such that only events of sufficient importance trigger an attention switch and the resulting distraction. Suitably, MMN and P3a amplitude appear to correlate with some quantification of the surprisingness or perceived importance of a deviant stimulus, increasing with the discriminability (103–105), task-relevance (104) and rarity of the deviant sound (106–108). Further, both components show attenuation with repetition consistent with a reduction of perceived stimulus importance as it is becomes familiar and a subsequent filtering of this information (51, 109–111).

Importantly, MMN and P3a are dissociable—MMN is not invariably followed by a P3a nor do their amplitudes reliably correlate (112–114). As a result, not every deviant event results in an attention switch (115, 116). Instead, it is more likely that the amplitude of MMN must exceed some variable threshold signifying its likely importance for behavior for the involuntary redirection of attention indexed by P3a to occur [e.g., MMN amplitude increases with deviant rarity (19, 117)]. These features serve the adaptive processing of new events—the ability to detect and direct attention for rapid evaluation, and subsequent habituation of this response in order to conserve resources once the stimulus is adequately assessed.

### Scalp Topography and Brain Networks

The distinct relationship of MMN to both sensory memory and attention gives legitimacy to a dual-generator model of MMN generation. Näätänen and Michie (118) first noted the large MMN amplitudes observed at temporal and frontal sites as indicative of two generators likely to be separately responsible for pre-attentive change detection and directing neural resources toward the change (i.e., attention) as per the previously assumed functions of these respective cortices. Separate temporal and frontal generators have been consistently identified using various source localization methods [e.g., (24, 90, 91, 119)]. More recently, dynamic causal modeling (DCM) has repeatedly favored a network of hierarchical cortical sources comprising the primary auditory cortex (A1), superior temporal gyrus (STG), and inferior frontal gyrus (IFG), as will later be discussed in detail (120–122). Cumulative observations have built a strong case for the early suggestion that these frontal and temporal components are differentially responsible for these sensory memory comparison and attention allocation functions respectively (72, 118).

A temporal generator for MMN is localized in primary auditory cortex, and is considered the primary generator responsible for MMN elicitation [(46, 123); see (89, 101) for reviews). This temporal contribution was first observed in magneto-encephalogram (MEG) studies identifying an equivalent current dipole on the supratemporal plane of the auditory cortex [(46, 123); see (89) for a review], and subsequent support has been accumulated across electrophysiological, hemodynamic, animal, and lesion studies [see (44, 101) for reviews]. This generator is believed to be responsible for the sensory memory component of MMN elicitation, given its direct receipt of sensory input and unique sensitivity to stimulus features. Temporal activation systematically increases with the degree of deviation on a single given dimension (91, 119), shows additivity in the case of multiple deviant features (124) and is impaired under increasing competition for resources when deviants are present across multiple sound streams (125). The precise area of activation within the supra-temporal cortex is modality-specific, showing variation based on deviant type (24) and tone complexity (57).

An additional source in prefrontal cortex has been proposed to be uniquely sensitive to the assumed relevance of the stimulus for behavior and redirection of attention toward this change. Frontal activation during MMN production was first identified in scalp current density maps (24) and subsequently confirmed in positron emission tomography [PET; (126, 127)], MEG (128), fMRI (90, 91, 129, 130), and optical imaging studies (131). Both frontal lesions (33, 132) and transcranial direct current stimulation of frontal sites (34, 133) have been associated with a general attenuation of MMN amplitude, highlighting this generator as a necessary contributor to adequate MMN production. Where temporal activation is highly sensitive to specific stimulus features, activity at frontal sites appears more reliant on an overall evaluation of global stimulus relevance which occurs upstream of initial sensory discrimination processes (91, 119, 124, 134). Consistent with this, activation follows a rostro-caudal gradient comprised of an “early MMN” component in the STG and a latter component in the IFG (95, 119, 135). This frontal component is believed to be responsible for the proposed “attention switch” toward the deviant stimulus, on the basis that it shows the same right-hemisphere asymmetry observed in the fronto-parietal network underlying spatial attention and orienting (136–139). While the literature emphasizes these two distinct temporal and frontal contributions to MMN generation, it is important to acknowledge that numerous source analyses, dipole models, and depth recordings in both human and animal studies reveal that these contributions occur within a complex network of activation including sub-regions comprising both temporal and frontal sources [see (140) for a review].

The placement of these generators within a hierarchically organized system has led to discourse around whether MMN generation should be considered a purely bottom-up process [i.e., initiated in lower-level, pre-attentive, sensory cortices with a processing cascade to increasingly higher (more frontal) areas], or may be subject to a top-down modulation [i.e., higher-order (more cognitive) processes originating in frontal cortices]. Early observations supporting the involvement of dual generators suggested that the initial activation of lower-level, temporal areas preceded any input from higher order regions [e.g., (91, 95, 141)]. Observations of impaired behavioral task performance during presentation of non-attended deviants even in the absence of deviant awareness suggests that this is indeed the case (142). However, MMN has also shown an early permeability to top-down effects which supports the reciprocity of these components. For example, explicit knowledge of the global sound sequence will determine whether MMN is elicited (35). An early top-down influence is also necessary to explain a shorter latency observed to omission deviants by Wacongne and colleagues (143). Further support for a concurrent top-down modulation stream is provided by observed effects of prediction and expectation discussed in later sections.

## Theoretical Accounts of MMN Generation

Naatanen (19, 72) acknowledged two possible theoretical interpretations for MMN—as either a legitimate memory-based ERP component or an artefact of differences in the adaptation of neurons tuned to the standard and deviant tones. Both perspectives offer an account of MMN which is substantial but non-exhaustive, and due to key differences are largely regarded as mutually exclusive. While the vast majority of studies into MMN since the 1970s have favored a memory-based account, there remains prominent discourse due to the explanatory power of the adaptation account and unanswered criticisms of memory-based perspectives (144, 145). These two lines of argument will be briefly expanded and the evidence for each reviewed, before the alternative possibility that these accounts could be unified as complementary components of MMN generation is presented.

### Memory-Based Hypotheses

The “sensory memory” or “memory mismatch” account views MMN as a distinct cognitive component of the auditory ERP which arises from the active comparison of current input with a memory trace for recently encountered sounds (58, 89, 102). MMN shares a number of characteristics with memory processes. The temporal window of integration for MMN elicitation is estimated between 7 and 20 s (142, 146, 147) which is consistent with the 5- to 20-s capacity previously observed for auditory sensory memory stores (56, 148). Meanwhile, elicitation of MMN to a previous deviant after a long period of intervening sound patterns suggests that multiple memory traces can lie dormant in longer-term memory and be reactivated when the stimuli are re-encountered (27, 32).

A popular explanation attributes MMN to a specialized change-detection or “feature-detector system” which actively analyzes and encodes physical features for storage in sensory memory (19, 53, 72). While it was initially asserted that the temporal scale of MMN necessarily separated any such system from the exogenous differences in neuronal activity which produce N1 to simple afferent changes, recent single-unit studies extending the time course of stimulus-specific adaptation (SSA) to as long as 60 s (149) suggest that a contribution to deviance detection at the cellular level may not necessarily be excluded (150). In any case, given the sensitivity of more frontal brain areas to longer-timescale information (151, 152), these observations are also consistent with the temporo-frontal network of activation previously discussed (24, 91, 95, 119). This memory-based account therefore considers the response to the deviant as the sum of the exogenous N1 response *and an additional* MMN component (53).

Following the observation of MMN to deviations of increasingly complex abstract rules [see (61) for a review], it was concluded that deviance detection cannot adequately rest on the direct comparison of current input to an afferent memory trace, and must instead involve a more sophisticated stored abstraction of the world constructed over longer time periods (12, 153, 154). The elicitation of MMN in the absence of any afferent basis for deviation highlighted a predictive component to deviance detection—discrepancy arises not from the features of sensory input per se, but rather the unfulfilled expectation of that stimulus. This is best evidenced by the elicitation of MMN by an unexpected sound omission (155), or violations of relative properties between sounds where discrepancy cannot be deduced by the simple comparison of absolute physical characteristics [e.g., a descending frequency interval within a consistently increasing-frequency scale (19, 156)]. This revised “regularity violation” or “model adjustment” hypothesis assumes that future input is actively extrapolated from the current memory store, and “absorbs” input consistent with this estimate, leaving only the remainder for processing (101, 153). Subsequently, this model is adjusted to better extrapolate future events (12), and some have argued that it is this maintenance of regularity representations which is the key function of MMN rather than the detection of deviance (154).

The model-adjustment hypothesis is furnished by the observed flexibility and sensitivity of MMN to recent exposure. MMN will be observed after as few as 2–3 repetitions of a new sound and show rapid reductions in amplitude as a new tone is repeated. The predictive representations are quickly formed, highly dynamic and incredibly sensitive to current contingencies in the world (157). This memory-based interpretation therefore assumes a distinct population of neurons capable of producing MMN which contribute to higher order perceptual-cognitive operations and embed a type of “primitive intelligence” within the auditory cortex (19, 53, 81, 101).

### The Adaptation Hypothesis

The adaptation hypothesis asserts the SSA of primary auditory cortex (A1) neurons tuned to the repeated standard sound would cause an attenuated N1 response much smaller than that produced by the “fresh afferents” tuned to the less probable deviant (144, 158). When compared, these responses would yield an additional negativity to the deviant sound in the 100- to 200-ms latency range of MMN. Take together, these ideas have led to the assertion by some that MMN represents a subtraction artefact rather than a distinct memory-based component. While this perspective accepts that long-latency and stimulus-specific A1 SSA may have a distinct and possibly specialized role in novelty detection (157), this is not commensurate with the functionally and anatomically distinct population of “comparator” neurons inferred by memory-based accounts (145). Rather, it is argued that A1 SSA is the single-unit correlate of MMN and the summed activity of A1 neurons is sufficient to account for the observed differences in the human ERP *in the absence of any higher-order operation*. The sensitivity of A1 SSA to multiple timescales—apparent in fast time constants of adaptation during short sequences and slow constants over long sequences similar to MMN—further demonstrated the ability of these simple, low-level mechanisms to mimic more sophisticated perceptual-cognitive effects (159, 160). On this basis, adaptation and memory-based hypotheses have been considered by some as mutually exclusive accounts of MMN production [e.g., (144, 145)].

Contention between the adaptation and memory-based interpretations of MMN is ongoing, given the outstanding criticisms and shortcomings for both hypotheses. Memory-based perspectives use observed differences in the morphology, topography, and sensitivity of the N1 and MMN as evidence that MMN arises from a distinct cognitive contribution to the deviant response [e.g., (116, 161, 162)]. Yet, empirical support for this idea is weakened by criticisms of the extent to which these differences reflect a pure measure of deviance, the absence of any direct evidence for the proposed population of neurons capable of this higher-order change detection, and a lack of consistent support from animal and intracranial studies [e.g., (163–165); c.f. (166)]. The adaptation account rests on conflicting studies which have failed to identify any unique change-specific activation in the response to a deviant sound [e.g., (167, 168)] and convincing demonstrations of neural refractoriness to produce MMN-like responses [e.g., (144)] with higher-order sensitivities [e.g., (160)]. These studies argue that the lower-level attributes of sensory neurons are in fact sufficient to account for any differences that might be observed in the response to the deviant sound including in both amplitude and topography [e.g., (169)].

However, neural adaptation also falls short of an exhaustive account of all aspects of MMN amplitude modulation. Adaptation fails to account for the large MMN elicited by repetition deviants (170–172), stimulus omissions (63, 64, 143), and unpredicted versus predicted deviant tones (173, 174). Further, additional negativity observed to a deviant tone using the previously discussed “controlled standard” or “many standards” paradigms reveals a modulation of responses that cannot be attributed to SSA (36, 161, 175). Here, the difference waveform is generated by the subtraction of the response to the same tone when separately encountered within a block of equiprobable control tones, necessarily ruling out any effect of physical differences in stimuli or the rate with which it was previously encountered. More recently, this paradigm has been widely adopted among animal studies and has provided compelling support for populations of cells along the auditory hierarchy which demonstrate genuine change detection as opposed to simple SSA (176–178). An additional important contribution to resolving such issues is strong evidence that the dominant influence over whether MMN is observed is reliant on transitional probabilities and not probability itself—a result inconsistent with stronger adaptation for frequent than for infrequent sounds (179).

### The Predictive Coding Framework

More recently, a memory-based account of MMN generation has been formalized within the framework of predictive coding, a general theory of brain function which frames perception as the integration of sensory input with predictions about the likely characteristics of this input based on prior exposure (4, 180–182). From this perspective, MMN is considered the neural substrate of “prediction error” elicited when there is a discrepancy between current input and the prediction [such as when an unexpected deviant sound is encountered (3, 12, 183)]. Prediction error is a proxy for surprise which serves to (1) alert the system and direct neural resources toward the unexpected event [consistent with (23, 72, 81, 101)] and (2) trigger an update to the existing “prediction model” to integrate discrepant input [consistent with (12, 153, 184)]. Critically, predictive coding models rest on the assumption that neural populations dynamically adjust responding to minimize prediction error and optimize predictions over repeated exposure to a stimulus (3, 120, 121). By specifying parameters for model updating and a neurobiological scheme in which they might be implemented, predictive coding allows for structured models in which memory-based mechanisms can be tested.

Predictive coding models MMN generation within a cortical hierarchy which uses reciprocal forward and backward connections to integrate input with predictions (3, 184). Afferent input is communicated “bottom-up” *via* forward connections from sensory cortices, while predictions about this input are communicated “top-down” *via* backward connections from higher brain areas (4, 181). The prediction error quantified by MMN is determined by the relative strength of intrinsic (within-area) and extrinsic (between-area) connections to modulate responding *via* changes in synaptic efficacy and sensitivity (3, 184). Higher cortical areas work to “explain away” predicted input *via* top-down suppression of error units to redundant sounds, while lower level areas feed forward an exuberant bottom-up prediction error to any aspects of input which are not predicted (3, 181).

Computational models have had some success in explaining the activity of neural populations during predictive coding *via* empirical Bayesian methods of prediction generation and updating (3, 180, 185). Empirical Bayesian approaches involve estimation of posterior probability based on a prior probability distribution derived from observation. This specific approach is in contrast to standard Bayesian approaches where the prior distribution is pre-defined. Sensory information is often limited, and a Bayesian perspective affords computations by which the brain effectively fills in the gaps for perception (17, 186). To maximize the accuracy of these estimates the “internal model” (180) or “prediction model” (12) is specified by Bayesian estimates of likelihood (probability that the given sensation would be produced by a particular cause) and a prior (the probability that cause would be encountered), which is based on previous observation and continually updated in line with the current observation. Where prediction error occurs, these estimates are updated to consistently reflect the most recent state of the world.

The relative influence of prediction errors at any one time is further weighted by estimates of “confidence” (12) or “precision” (3) which reflect the expected accuracy of the prediction model and are embodied in the post-synaptic sensitivity or gain of populations encoding prediction error units (122, 185, 187, 188). The more accurate a prediction has been in the recent past the stronger top-down suppression and less permeable it is to immediate revision following prediction error. Conversely, in more variable and unpredictable environments, larger prediction errors will impact the prediction model which is more readily adjusted. This variable weighting of observed data is further represented as a hierarchical implementation of Bayesian methods, where the estimated probability is derived from estimates of several inter-dependent values. This updating of stored representations over multiple encounters of a stimulus, referred to as perceptual learning, shares commonalities with more general optimal learning algorithms such as the Kalman filter (189). Empirical Bayesian methods of estimation provide constraints for predictive coding which can feasibly be transcribed on neuronal populations to ensure the optimal minimization of perceptual uncertainty at all levels of the cortical hierarchy [see (185) for discussion].

A hierarchical Bayesian model of predictive coding as described above is theoretically sufficient to account for numerous aspects of change detection including enhanced gamma-band (190, 191), blood-oxygen-level-dependent (91, 119), and electrophysiological responses to a deviant sound (3, 121, 143, 183), the prediction-dependent suppression of responses to a standard sound (192–194) and reductions in MMN onset latency with repetition as a result of top-down facilitation (87, 193). The proposed hierarchical structure is in accordance with a temporo-frontal network of MMN generation [e.g., (24, 95)] where more frontal areas display longer latencies of activation [e.g., (195)] and there is a disinhibition of responses to the standard when these frontal areas, responsible for top-down suppression of error signals, are lesioned (33, 196, 197).

At the neural level, the N-methyl-D-aspartate (NDMA)-dependent plasticity of cortical connections provides a feasible basis for predictive coding, given that NMDA receptors have been implicated in both synaptic learning and MMN generation [(3, 185, 198, 199); see (171) for a recent model] and MMN itself has been proposed as an index of NMDA-receptor (NMDAR) function (200). More recently, DCM has provided more direct empirical support for predictive coding by demonstrating that changes in cortical connectivity during deviant versus standard sound processing is best explained by a hierarchical model with nodes in primary auditory, temporal, and prefrontal cortices comprised of both forward (bottom-up), backward (top-down) and lateral (within-area) connections (3). Taken together, these results provide cumulative support for a hierarchical generative model of MMN generation, where synaptic plasticity between a hierarchy of brain areas is used to generate and optimize predictive inferences about sensory input to facilitate perception in line with empirical Bayes. MMN is a functional neural substrate of prediction error which reflects a synergy of smaller-scale sensory processes within and between cortical areas in order to construct higher-order memory representations.

### Uniting Predictive Coding and Adaptation Accounts

Computational models of predictive coding also have the capacity to unify the conflicting adaptation and memory-based accounts of MMN generation (3, 121). Predictions are modeled as adjustments of the post-synaptic sensitivity of intrinsic and extrinsic connections which are optimized over repeated exposures to a stimulus to minimize prediction error. Reduced sensitivity at the neuronal level within these models resembles the SSA of A1 neurons which forms the basis for the adaptation hypothesis [e.g., (201)]. DCM studies have consistently demonstrated that a network comprised only of intrinsic connections, representing an adaptation-only account, is inferior to more distributed network models in explaining MMN generation (3, 120, 122). These computational models therefore support the earlier suggestion that in fact neither of the competing accounts alone are sufficient (3) and that the explanatory power of one account does not necessarily render the other obsolete (53).

One criticism of the adaptation account has been the interchangeable use of terms relating to active adaptation and passive refractoriness in the MMN literature which lead to interpretive error (202). The predictive coding models constructed by Garrido (120–122) emphasize the purposeful adjustment of the post-synaptic sensitivity or gain of error units [i.e., what O'Shea (202) argues is true adaptation, as opposed to passive “sluggish” refractoriness] as crucial to optimizing predictive processes. This is consistent with a conceptualization of MMN as reflecting a compound mismatch process, of which both a sensitized response to a deviant sound and suppression of response to a repeated sound are necessary components and are adequately captured by predictive coding (203). A move toward a unified account of MMN is also being observed in animal models. A similar sensitivity to deviant probability and degree of difference shown by auditory SSA and MMN has led to the suggestion that auditory SSA likely represents an early single-neuron correlate in auditory cortex which is necessary but not sufficient to explain the longer-latency MMN response arising from a compound of primary auditory and higher cortical areas (204–206). This is consistent with more recent research delineating the reduction of early (40–60 ms) latency components with repetition which is presumed to arise from SSA, from that observed in later (100–200 ms) latency components which is exclusively reliant on prediction (194).

## A First-Impression Bias in Auditory Processing

While the utility of MMN rests on this ability to flexibly represent up-to-date probability statistics, a growing body of research suggests that MMN amplitude modulation does not always consistently reflect environmental change. *First-impression* or *primacy bias* refers to the novel observation that MMN amplitude to two tones will show differential patterns of modulation over the course of a changing sound sequence based on their relative probabilities when first encountered at sequence onset. This lasting effect of initial learning on subsequent processing demonstrates that while MMN amplitude may be highly dynamic, it can be biased by prior experience. These more novel studies therefore suggest that MMN does not necessarily provide a veridical representation of the current state of probability statistics at any given time.

### Experience Matters: An Order-Driven Effect

**Figure 1:**
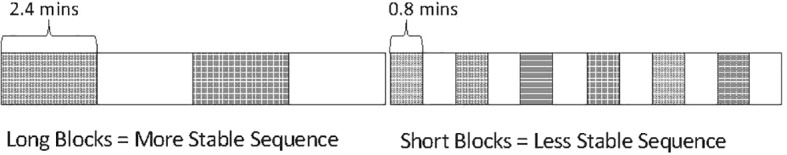
Representation of original multiple-timescale sequence. Depiction of sound sequence design in the multiple-timescale paradigm used by (8). Dark blocks represent “first context” blocks where one tone is presented with standard probability (p = .825) and the other tone with deviant probability (p = .125). Light blocks represent “second context” blocks where these tone probabilities are reversed (i.e., the originally standard tone becomes the deviant and the originally deviant tone becomes the standard). Sound sequences were created using these block types with different lengths, forming a “slow change” sequence consisting of 2.4-min blocks, and a “fast change” sequence consisting of 0.8-min blocks.

The first-impression bias is revealed and studied using an augmentation of a traditional oddball sound sequence termed the *multiple-timescale paradigm*, depicted in **Figure 1**, where two tones alternate in the role of standard and deviant across two block types (represented as dark versus light boxes in **Figure 1** and hereafter referred to as first and second context) at different rates between sequences. The term multi-timescale reflects the fact that there are visibly both local regularities (within the blocks) and longer-term regularities (in regular block length).

**Figure 2:**
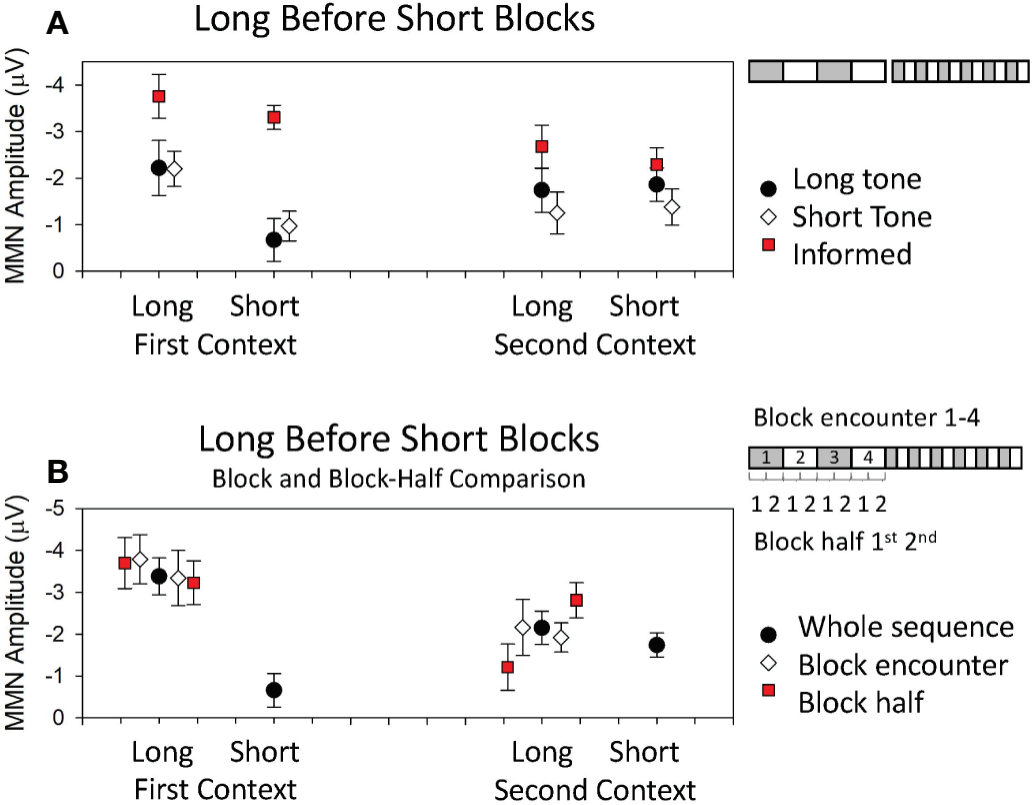
Data from published multi-timescale studies. Detailed descriptions of the studies are provided in text. **(A)** Mean MMN amplitudes obtained from studies using a long block sequence before short block sequence. Black dots and white diamonds represent mean MMN amplitudes obtained in (15) where participants heard the sequences first with the long tone as the deviant in the first context and then the short tone as the deviant in the first context. The red squares represent mean MMN amplitudes obtained in (207) when the long tone was the deviant in the first context, but participants were first informed about the structure and the composition of the sequences before hearing them. **(B)** Mean MMN amplitudes obtained in (208) where the long tone was the deviant in the first context. Data show amplitudes obtained from the whole sequence (black dots), the first and second encounter with a given long block context (white diamonds), and the early and later half of the long blocks (red squares).

Traditional accounts of MMN as a highly dynamic confidence-weighted error signal might lead us to suppose that MMN amplitude will show a consistent and parametric increase with the stability of current patterns which rapidly adjusts when these patterns change. It follows that MMN should therefore be larger in blocks of longer duration for both first and second context blocks. The multiple-timescale paradigm has revealed that MMN to the two tones throughout the course of the sequence remains differentially sensitive to the stability of current patterns based on probabilities of these two tones at sequence onset. MMN was only larger in longer, more stable (2.4 min) blocks compared to shorter, comparatively less stable (0.8 min) blocks for the tone which was initially in the role of deviant [i.e., in first context blocks (8)]. MMN to the tone which was deviant in the second context (i.e., MMN to the tone which initially occurred with standard probability, after it became a deviant; in the second context) did not differ in amplitude across periods of relatively longer or shorter pattern stability (8, 15). To illustrate these effects, data from (15) are reproduced in **Figure 2A** where the black dots depict MMN amplitude to deviant that were 60 ms in duration among common tones that were 30 ms in duration. The white diamonds in **Figure 2A** depict the MMN amplitude to deviants that were 30 ms in duration among common tones that were 60-ms long. The data on the left depict the MMN amplitudes when these sounds were deviant in the grey blocks of **Figure 1** (i.e., the first context), while those on the right depict the MMN amplitudes to the same tones when they were deviant in the white blocks of **Figure 1** (i.e., the second context). It is clear from **Figure 2A** that MMN is only larger in longer blocks for sounds that were the deviant encountered in the first heard context, irrespective of tone feature; that is, this is an order-driven effect. MMN in these sequences, under these experimental conditions, did not provide a veridical representation of probability statistics in both contexts as traditional accounts would predict. All of the data presented in **Figure 2A** was acquired from participants naïve to the sequence in that they had not participated in any previous multiple-timescale study and did not know about the sequence structure. Each participant was told that brain activity being measured occurred automatically and was best measured when participants ignore the sound and focus on the task of watching a DVD with subtitles. This finding violates the idea that the confidence weightings which underlie MMN generation are solely governed by current (local) probability statistics.

In a subsequent study designed to investigate the mechanisms underlying these order effects, the data from within blocks was divided to look separately at what happened to MMN early in the blocks when a local model had just been established (1^st^ half), versus later in blocks once the model had been stable for a while (2^nd^ half, see graphic in **Figure 2B** right). When examining MMN amplitude change within blocks, the differential effect of stability in first and second contexts was most pronounced in the first half of blocks immediately after tones change roles and effectively “washed out” such that there was no difference between MMN to the two tones as deviants when comparing the latter half of sequence blocks (209). The first-impression bias therefore appeared to arise from some order-driven bound on the accumulation of predictive confidence which was formed at sequence onset and skewed pre-attentive sensory processing toward the confirmation of what was first learnt until sufficient evidence to override this first learning was accumulated. This difference between block halves has been repeated in subsequent studies and an example of these half-effects for the longer blocks is presented in **Figure 2B** in data reproduced from (208). In **Figure 2B**, the black dots depict MMN to deviants that is calculated from all relevant blocks of the sequences and the red squares depict the MMN amplitude when calculated from the early period of the two long blocks (1^st^ half) to the left of the black dots, and from the later period of the two long blocks (2^nd^ half) to the right of the black dots. It is clear in **Figure 2B** that MMN to the deviants in the first context are large throughout long blocks of the sequence, while MMN to deviants in the second context long blocks start smaller and amplitude increases as the block continues. These differences over block half contrast the relative equivalence of the MMN amplitudes evident in averages taken from the first and second encounter of the long blocks (see graphic **Figure 2B**, right). In **Figure 2B**, the white diamonds depict MMN amplitude for the first block encounter of the first and second context presented to the left of the black blocks, and that to the second block encounter for each context is presented to the right.

The novelty of this first-impression bias generates a series of important questions that must be addressed in service of a comprehensive understanding of the form and function of MMN and its contribution to perceptual-cognitive processes. The first requires establishing to what extent this modulation is attributable to temporal order effects over and above any other characteristic of the sound sequence (e.g., the physical properties of the tones). Should the observed effect be confidently attributed to tone order, there follows the question of to what extent it generalizes across sequence structures, tone types, and deviations. As noted, **Figure 2A** is derived from sequences in which the two sounds differ in duration (30 and 60 ms) and the same pattern of MMN amplitude modulation is obtained for the two block types whether the long tone or the short tone is rare in the first context (i.e., it is order-dependent not feature-dependent [(15), see also (210)]. Certainly, there is also evidence that similar modulation patterns can be observed using frequency deviants (7) and spatial deviants (208) offering support to the notion that it is a general order-driven effect.

Order-driven effects on MMN amplitude have elsewhere been observed in a study of shorter sound sequences where tones of different frequency switched roles as standard and deviant only once (160) and where authors attributed this to longer-timescale adaptation effects exerting bottom-up influence on ERP amplitudes. The study was designed to replicate earlier work demonstrating the impact of long-term SSA of single neurons on standard and deviant ERPs (159). The authors concluded that an initial “suppression” of MMN amplitude to a deviant with a long history of repetition after tones change roles could reflect the existence of similar SSA mechanisms occurring over multiple timescales in the human auditory cortex simultaneously (160). Longer timescale adaptation was demonstrated lasting up to 10 s, alongside a faster adaptation time constant of 1.5 s to local patterning, and appeared to show similar development to that seen in single-neuron studies of the cat auditory cortex (159). Costa-Faidella and colleagues (160) further demonstrated the successful prediction of MMN amplitude modulations through a linear model of local and global adaptation effects and argued that order-driven effects such as those observed by Todd and colleagues (8) can arise from basic, bottom-up properties of the auditory system. One difference between the two studies was the repeated alternation of tone arrangements in (8). In **Figure 2B**, the breakdown of data gives us an opportunity to examine the response to a deviant sound that has never been common (block 1, graphic on the right), versus the response to the same sound when it has just been common (block 3, graphic on the right). Based on SSA effects, we would assume the response to deviants in block 3 to be much smaller than in block 1, and the difference between deviants in blocks 3 and 4 to be diminished relative to differences between blocks 1 and 2. This is clearly not the case in data represented by the white diamonds.

Interestingly, the MMN amplitude modulations that occur are very different if a group of participants are first shown **Figure 1** diagram and told about the sequence structure before being given the same instruction about the automaticity and ignoring the sounds while watching a DVD with subtitles. Under these “informed” conditions, the long > short block MMN amplitude modulation is absent for both contexts (see red squares, **Figure 2A**). Finally, if these same sequences are heard by participants who are performing a more cognitively demanding visual task the results are different again with MMN amplitude in long > short blocks for both contexts (207).

Every dataset displayed in **Figure 2** emerged from the same two sounds with the same local probabilities (a 60 and 30 ms, 1,000 Hz pure tone at p = 0.875 when common and p = 0.125 when rare), and yet the MMN amplitude modulation patterns are quite different. This compilation of data illustrates that the MMN amplitudes produced to these simple sequences are highly dependent on the longer-term sequence structure, and the learning environment in which they are heard. This pattern occurs in a way that seems difficult to account for by SSA—at least not SSA considered to arise as an inevitable suppression of response based on a recent history of frequent presentation. In the following section, we explore a more complex account of order-effects that might accommodate these puzzling observations.

### A Hierarchical Bayesian Perspective

The first-impression bias can be captured by the implementation of predictive coding within a hierarchical Bayesian learning scheme, where the processing of sensory input at each level is modulated by top-down priors which are weighted by estimates of confidence or accuracy and based on information collected over longer time periods (3). The influence of these backward connections embodies predictions, enforcing the suppression of prediction error units to a predicted sound in a manner that reflects expected precision based on the previous stability or predictability of the environment [i.e., gain control (211)]. The interpretation offered for the bias is that in the absence of a pre-existing prior for the two sounds at sequence onset, there is a rapid accumulation of precision for the initial deviant as rare and informative and the initial standard as redundant and uninformative (8, 212). These high confidence weightings equate to strong top-down predictions which are highly effective in suppressing prediction error to the uninformative standard tone. When tone roles change, the ability to accept this initially uninformative tone as a potentially important deviant is then limited as this highly suppressed error signal has a minimal impact on learning rate, leading to marked differences in how the two tones are processed as deviants.

The differential effects observed to the two tones are dominated by modulations of the deviant ERP, suggesting that it is principally the processing of surprise rather than redundancy which is biased [however, see (213) for more subtle order-dependent modulation of the standard ERP]. Modulation of response to the deviant tone is consistent with predictive coding, where precision or gain is specifically reflected in how effectively prediction error to the deviant sound is suppressed. While neural adaptation has previously been shown to influence MMN (160), this explanation alone is insufficient to explain why bias patterns persist throughout the duration of the sequence. Under this account, it would be expected that a similar suppression would be observed to subsequent presentations of the first context blocks after the first deviant has spent a period of time in the role of standard—**Figure 2B** shows that this did not explain the data in this case. Neural adaptation would also struggle to account for how, when a prior exists (informed condition, red squares, **Figure 2A**), this difference in weightings for the two contexts does not occur (207). These modulation patterns may instead be linked to some form of higher-order representation which is effectively re-activated each time the first context block is re-encountered. Prediction models have previously been shown to have a degree of context specification, given that no tone can behave as both standard and deviant in a given context (214). In this way, predictive coding could offer a sufficient mechanistic explanation of the first-impression bias as evidence for the influence of tightly held, top-down representations of sounds on future sound processing which involve some form of higher level, semantic categorization. Accordingly, the different data acquired from informed participants (**Figure 2A**) may indicate that foreknowledge enables more flexibility in model updating as a function of knowing in advance that category memberships will change.

### Multiple Timescales of Statistical Learning

The presence of regular block lengths is central to the observed patterns of bias. The differential modulation patterns to first and second deviant tones do not occur if the four longer blocks are intermixed with the 12 shorter blocks such that there is no predictable longer term temporal structure (213). In this study, sequences always started with a long block of the 60-ms tone as first deviant, and blocks always alternated tone probabilities, but there was no regularity in the block alternation rate. Under these circumstances, the MMN amplitudes were larger with longer local regularity for both contexts. However, larger MMN amplitudes for longer blocks are not observed if the four longer blocks occur after a regular pattern of twelve shorter blocks. In this case, MMN amplitudes in longer blocks are either equivalent throughout the entire sequence for both contexts (215), or indeed larger for the shorter blocks than the longer blocks for the first context (14). It has therefore been suggested that high precision associated with the first context remains influential if the longer-term environment is predictable, but will be lost if the environment changes in an unexpected way (e.g., blocks are shorter or longer than expected). This explains an expected short < long block pattern for first-context if the long blocks are first, but counteracts this pattern if the long blocks are second.

The importance of first-impressions is perhaps even more convincingly demonstrated in a recent three-tone multiple-timescale sequence (37). In this study, three sounds were arranged in blocks where two were equally common and one was rare, and the probabilities rotated creating three different block types (i.e., probabilities, A < B = C, B < A = C, C < A = B). The sequences included two of each block type with three versions—one starting with A < B = C, one with B < A = C and one with C < A = B. While MMN was generated to the rare tone in each block of all sequence arrangements, the MMN generated to any deviant in any block was always significantly smaller if the sequence began with two common sounds with the highest spatial separation (90° left or right). In other words, despite equivalent sound compositions within blocks inside the different sequences, the auditory system assessed the configurations in which the two common tones were adjacent in space (within the three locations used) as less volatile compared to when they were highly separate. However, remarkably, the effect of this increase in volatility was *only evident* when the more volatile environment was encountered at the beginning of the sequence. A volatile first-impression at sequence onset led all deviance-related responses to be significantly lower in amplitude for the ensuing 12-min period.

### Implications for Cognitive Neuroscience

First-impression bias has been interpreted to reflect a sensitivity to information collected across multiple timescales that alters model updating [e.g., (7, 15)]. High confidence in initial tone roles leads to slowed accumulation of confidence for the new roles once these change, but it appears to be prevented by sequence foreknowledge (207). At a local level, the inversion of tone probabilities overwrites the current prediction model, but some memory of the first impression remains and seems to be reactivated when the initial block structure is encountered again (15). This may suggest, for example, that the reversal of probabilities becomes treated as a temporary departure from the initial prediction model, revealing the ability of the auditory system to maintain information beyond even the longest 30-s temporal limit previously proposed to apply to MMN (216).

The interpretations offered above are controversial in their opposition of much of what is considered “known” about the mechanisms and meaning of MMN. Instead, they appeal to more sophisticated models of learning which have gained favor elsewhere. The Hierarchical Gaussian Filter (HGF), for example, provides a revision of Rescorla and Wagner's (217) model of associative learning where rather than learning rates being directly proportional to error, they are hierarchically weighted relative to various degrees of uncertainty that more closely imitate a stochastic real-world environment (218, 219). The HGF assumes that learning proceeds in a Bayes-optimal fashion similar to the hierarchical precision weightings described above. In the same way that a single repetition of a sound is not sufficient to elicit MMN (220), this ensures that new learning is not triggered by chance fluctuations. These similarities may speak to the generality of these fundamental learning mechanisms to perception. The first-impression bias therefore has potential not only to advance our understanding of MMN as a neural marker for basic brain processes but could itself as a tool for probing more complex neural computations.

Order effects on MMN are part of an evolving literature encouraging a revisiting of assumptions about the processing underlying MMN. Studies that emphasize the centrality of transitional probability rather than probability per se [e.g., (179)] are consistent with the notion of future state predictions being a priority for sensory information processing (3, 184) even in task-independent listening where sound has no direct implications for behavior. The order effects we have reviewed here prompt a reconsideration of the timescales over which predictive processing is operating. The critical influence of volatility estimates demonstrated here necessarily reflects longer-timescale attributes of sequential sound presentation. In conclusion, these observations introduce the potential for new applications of MMN as a tool in cognitive neuroscience and expand the questions and interpretations that might be put forward in its use to explore “sensory information processing abnormalities in schizophrenia and related neuropsychiatric disorders”.

### Application in Clinical Cognitive Neuroscience

MMN, in general, is a useful candidate for exploring basic cognitive neuroscience and psychopathology. First, MMN has good individual test-retest reliability and sensitivity to inter-individual differences in a number of domains (71). Given that MMN elicitation relies on the detection of discrepancy between a deviant and standard sound it thereby provides a means to measure individual auditory discrimination ability (18). Studies have demonstrated the use of MMN to infer individual performance on related processes ranging from memory trace formation (82, 221), auditory stream segregation (174) and regularity extraction (79). Variation in MMN has also been used to track intra-individual changes, which can be useful in both general observation and the assessment of intervention effects. MMN has previously been used to measure the effects of pharmacotherapy [e.g., (222)] and auditory training [e.g., (223, 224)] and holds promise as an endophenotypic marker of dysfunction in certain conditions including pathological aging [e.g., (225, 226)] and psychotic disorders [e.g., (222, 227, 228)]. Another advantage of MMN as a clinical measure is that the change detection process underlying MMN appears to be initiated at least in part in an early, pre-attentive level of the cortical hierarchy (19). MMN elicitation does not require a participant to consciously attend to stimuli and provides a means to study a wide range of cognitive operations in populations where attention or motivation may be lacking, such as children or the very impaired (38, 229). In both clinical and healthy populations, MMN can be used to elucidate automatic or pre-attentive cognitive processes and their downstream effects on voluntary and controlled processing (230).

Variables listed in **Table 1** include many that can be experimentally manipulated to investigate a rich array of questions in clinical groups, and the potential to explore the influence of multiple-timescale patterning within sequences and order-effects of volatility are now added inclusions in this suite. The caveat highlighted here however is that MMN is elicited within a specific learning environment created by the experimenter. The behavior of the inferential system under investigation will likely be nuanced by what predictions the system is attempting to optimize [see (213) for discussion]. Even within studies of schizophrenia, arguably, the most mature clinical application of MMN, there are inconsistencies in studies attempting to identify the core anomaly in the underlying system. Although reduced amplitude MMN is a highly replicable finding with large effect sizes [e.g., (231)], evidence for whether this reflects purely an impaired response to deviation (232, 233) or impaired encoding of regularity as well as deviance (26, 234) is mixed. Similarly, attempts to localize the deficit within the inferential network yield inconsistent findings [see (235, 236, 237) for reviews]. While deficient formulation and encoding of valid predictions is considered a central feature of psychotic phenomena (238), and auditory inference is an excellent methodology in which to study the integrity of valid predictions, a full mechanistic understanding of the underlying causes remains elusive. Ultimately, a deeper understanding of the differences within an inferential system remains reliant on a deeper understanding of the system itself, and here, we propose that a consideration of timescales of learning adds a potentially informative consideration in understanding how paradigms might differ, and how group differences might differ across paradigms.

## Author Contributions

KF completed the first draft of this document. JT edited the document and added significant contributions to content.

## Funding

KF acknowledges receipt of Australian Postgraduate Award scholarships. This research was supported by funds provided by the National Health and Medical Research Council of Australia (APP1002995).

## Conflict of Interest

The authors declare that the research was conducted in the absence of any commercial or financial relationships that could be construed as a potential conflict of interest.

## References

[1] Shelley AM Ward PB Catts SV Michie PT Andrews S McConaghy N Mismatch negativity: an index of a preattentive processing deficit in schizophrenia. Biol Psychiatry (1991) 30(10):1059–62. 10.1016/0006-3223(91)90126-7 1756198

[2] Bregman AS Auditory scene analysis: the perceptual organization of sound. Cambridge, MA: The MIT Press (1990). Retrieved from https://books.google.com.au/books/about/Auditory_Scene_Analysis.html?id=jI8muSpAC5AC&redir_esc=y.

[3] Friston K A theory of cortical responses. Philos Trans R Soc Lond B Biol Sci (2005) 360(1456):815–36. 10.1098/rstb.2005.1622 PMC1569488 15937014

[4] Rao RPN Ballard DH Predictive coding in the visual cortex: a functional interpretation of some extra-classical receptive-field effects. Nat Neurosci (1999) 2(1):79–87. 10.1038/4580 10195184

[5] Frost JD Winkler I Provost A Todd J Surprising sequential effects on MMN. Biol Psychol (2016) 116:47–56. 10.1016/j.biopsycho.2015.10.005 26493340

[6] Todd J Provost A Whitson L Mullens D Initial uncertainty impacts statistical learning in sound sequence processing. J Physiol Paris (2018) 389:41–53. 10.1016/j.neuroscience.2018.05.011 29782815

[7] Todd J Heathcote A Whitson L Mullens D Provost A Winkler I Mismatch negativity (MMN) to pitch change is susceptible to order-dependent bias. Front Neurosci (2014) 8. 10.3389/fnins.2014.00180 PMC4069482 25009462

[8] Todd J Provost A Cooper G Lasting first impressions: a conservative bias in automatic filters of the acoustic environment. Neuropsychologia (2011) 49(12):3399–405. 10.1016/j.neuropsychologia.2011.08.016 21872613

[9] Czigler I Visual Mismatch Negativity. J Psychophysiol (2007) 21(3):224–30. 10.1027/0269-8803.21.34.224

[10] Näätänen R Paavilainen P Rinne T Alho K The mismatch negativity (MMN) in basic research of central auditory processing: a review. Clin Neurophysiol (2007) 118(12):2544–90. 10.1016/j.clinph.2007.04.026 17931964

[11] Picton TW Alain C Otten L Ritter W Achim A Mismatch negativity: different water in the same river. Audiol Neuro-Otol (2000) 5(3–4):111–39. 10.1159/000013875 10859408

[12] Winkler I Interpreting the mismatch negativity. J Psychophysiol (2007) 21(3–4):147–63. 10.1027/0269-8803.21.34.147

[13] Garrido MI Kilner JM Stephan KE Friston KJ The mismatch negativity: A review of underlying mechanisms. Clin Neurophysiol (2009) 120(3):453–63. 10.1016/j.clinph.2008.11.029 PMC2671031 19181570

[14] Mullens D Winkler I Damaso K Heathcote A Whitson L Provost A Biased relevance filtering in the auditory system: A test of confidence-weighted first-impressions. Biol Psychol (2016) 115:101–11. 10.1016/j.biopsycho.2016.01.018 26844870

[15] Todd J Provost A Whitson LR Cooper G Heathcote A Not so primitive: context-sensitive meta-learning about unattended sound sequences. J Neurophysiol (2013) 109(1):99–105. 10.1152/jn.00581.2012 23076102

[16] Geisler WS Diehl RL A Bayesian approach to the evolution of perceptual and cognitive systems. Cogn Sci (2003) 27(3):379–402. 10.1016/S0364-0213(03)00009-0

[17] Knill DC Pouget A The Bayesian brain: the role of uncertainty in neural coding and computation. Trends Neurosci (2004) 27(12):712–9. 10.1016/J.TINS.2004.10.007 15541511

[18] Näätänen R Gaillard AW Mantysalo S Early selective-attention effect on evoked potential reinterpreted. Acta Psycholog (Amsterdam) (1978) 42(4):313–29. 10.1016/0001-6918(78)90006-9 685709

[19] Näätänen R Attention and Brain Function. Hillsdale, NJ: Erlbaum (1992).

[20] Sams M Alho K Näätänen R Sequential effects on the ERP in discriminating two stimuli. Biol Psychol (1983) 17(1):41–58. 10.1016/0301-0511(83)90065-0 6626636

[21] Sams M Paavilainen P Alho K Näätänen R Auditory frequency discrimination and event-related potentials. Electroencephalogr Clin Neurophysiol (1985) 62(6):437–48. 10.1016/0168-5597(85)90054-1 2415340

[22] Näätänen R The mismatch negativity: a powerful tool for cognitive neuroscience. Ear Hear (1995) 16(1):6–18. 10.1097/00003446-199502000-00002 7774770

[23] Schröger E On the detection of auditory deviations: a pre-attentive activation model. Psychophysiology (1997) 34(3):245–57. 10.1111/j.1469-8986.1997.tb02395.x 9175439

[24] Giard M-H Perrin F Pernier J Bouchet P Brain generators implicated in the processing of auditory stimulus deviance: A topographic event-related potential study. Psychophysiology (1990) 27(6):627–40. 10.1111/j.1469-8986.1990.tb03184.x 2100348

[25] Schröger E Wolff C Attentional orienting and reorienting is indicated by human event-related brain potentials. Neuroreport (1998a) 9(15):3355–8. 10.1097/00001756-199810260-00003 9855279

[26] Baldeweg T Klugman A Gruzelier J Hirsch SR Mismatch negativity potentials and cognitive impairment in schizophrenia. Schizophr Res (2004) 69(2–3):203–17. 10.1016/j.schres.2003.09.009 15469194

[27] Cowan N Winkler I Teder W Näätänen R Memory prerequisites of mismatch negativity in the auditory event-related potential (ERP). J Exp Psychol Learn Memory Cogn (1993) 19(4):909–21. 10.1037/0278-7393.19.4.909 8345328

[28] Winkler I Näätänen R Event-related potentials in auditory backward recognition masking: A new way to study the neurophysiological basis of sensory memory in humans. Neurosci Lett (1992) 140(2):239–42. 10.1016/0304-3940(92)90111-J 1501786

[29] Winkler I Paavilainen P Alho K Reinikainen K Sams M Naatanen R The effect of small variation of the frequent auditory stimulus on the event-related brain potential to the infrequent stimulus. Psychophysiology (1990) 27(2):228–35. 10.1111/j.1469-8986.1990.tb00374.x 2247552

[30] Csépe V Karmos G Molnár M Evoked potential correlates of stimulus deviance during wakefulness and sleep in cat — animal model of mismatch negativity. Electroencephalography Clin Neurophysiol (1987) 66(6):571–8. 10.1016/0013-4694(87)90103-9 2438122

[31] Paavilainen P Jaramillo M Näätänen R Binaural information can converge in abstract memory traces. Psychophysiology (1998) 35(5):483–7. 10.1017/S0048577298970895 9715092

[32] Korzyukov OA Winkler I Gumenyuk VI Alho K Processing abstract auditory features in the human auditory cortex. NeuroImage (2003) 20(4):2245–58. 10.1016/j.neuroimage.2003.08.014 14683726

[33] Alain C Woods DL Knight RT A distributed cortical network for auditory sensory memory in humans. Brain Res (1998) 812(1–2):23–37. 10.1016/S0006-8993(98)00851-8 9813226

[34] Weigl M Mecklinger A Rosburg T Transcranial direct current stimulation over the left dorsolateral prefrontal cortex modulates auditory mismatch negativity. Clin Neurophysiol (2016) 127(5):2263–72. 10.1016/j.clinph.2016.01.024 27072099

[35] Sussman E Winkler I Huotilainen M Ritter W Näätänen R Top-down effects can modify the initially stimulus-driven auditory organization. Cogn Brain Res (2002) 13(3):393–405. 10.1016/S0926-6410(01)00131-8 11919003

[36] Jacobsen T Schröger E Horenkamp T Winkler I Mismatch negativity to pitch change: Varied stimulus proportions in controlling effects of neural refractoriness on human auditory event-related brain potentials. Neurosci Lett (2003) 344(2):79–82. 10.1016/S0304-3940(03)00408-7 12782332

[37] Todd J Frost J Fitzgerald K Winkler I Setting precedent: Initial feature variability affects the subsequent precision of regularly varying sound contexts. Psychophysiology (2020) 57(4):e13528. 10.1111/psyp.13528 31970811

[38] Näätänen R Kujala T Winkler I Auditory processing that leads to conscious perception: a unique window to central auditory processing opened by the mismatch negativity and related responses. Psychophysiology (2011) 48(1):4–22. 10.1111/j.1469-8986.2010.01114.x 20880261

[39] Näätänen R Simpson M Loveless NE Stimulus deviance and evoked potentials. Biol Psychol (1982) 14(1–2):53–98. 10.1016/0301-0511(82)90017-5 7104425

[40] Näätänen R Picton T The N1 wave of the human electric and magnetic response to sound: a review and an analysis of the component structure. Psychophysiology (1987) 24(4):375–425. 10.1111/j.1469-8986.1987.tb00311.x 3615753

[41] Renault B Lesevre N A Trial by Trial Study of the Visual Omission Response in Reaction Time Situations. In: . Human Evoked Potentials. Boston, MA: Springer US (1979) p. 317–29. 10.1007/978-1-4684-3483-5_21

[42] Näätänen R Picton TW N2 and automatic versus controlled processes. Electroencephalography Clin Neurophysiol Supplement (1986) 38:169–86. 3466775

[43] Patel SH Azzam PN Characterization of N200 and P300: selected studies of the Event-Related Potential. Int J Med Sci (2005) 2(4):147–54. 10.7150/ijms.2.147 PMC1252727 16239953

[44] Alho K Cerebral generators of mismatch negativity (MMN) and its magnetic counterpart (MMNM) elicited by sound changes. Ear Hear (1995) 16(1):38–51. 10.1097/00003446-199502000-00004 7774768

[45] Korzyukov O Alho K Kujala A Gumenyuk V Ilmoniemi RJ Virtanen J Electromagnetic responses of the human auditory cortex generated by sensory-memory based processing of tone-frequency changes. Neurosci Lett (1999) 276(3):169–72. 10.1016/S0304-3940(99)00807-1 10612632

[46] Sams M Kaukoranta E Hämäläinen M Näätänen R Cortical activity elicited by changes in auditory stimuli: different sources for the magnetic N100m and mismatch responses. Psychophysiology (1991) 28(1):21–9. 10.1111/j.1469-8986.1991.tb03382.x 1886961

[47] Pincze Z Lakatos P Rajkai C Ulbert I Karmos G Separation of mismatch negativity and the N1 wave in the auditory cortex of the cat: a topographic study. Clin Neurophysiol: Off J Int Fed Clin Neurophysiol (2001) 112(5):778–84. 10.1016/S1388-2457(01)00509-0 11336892

[48] Alho K Paavilainen P Reinikainen K Sams M Näätänen R Separability of different negative components of the event-related potential associated with auditory stimulus processing. Psychophysiology (1986) 23(6):613–23. 10.1111/j.1469-8986.1986.tb00680.x 3823336

[49] Novak GP Ritter W Vaughan HG Wiznitzer ML Differentiation of negative event-related potentials in an auditory discrimination task. Electroencephalography Clin Neurophysiol (1990) 75(4):255–75. 10.1016/0013-4694(90)90105-S 1691075

[50] Auksztulewicz R Friston K Attentional Enhancement of Auditory Mismatch Responses: a DCM/MEG Study. Cereb Cortex (2015) 25(11):4273–83. 10.1093/cercor/bhu323 PMC4816780 25596591

[51] Näätänen R Gaillard AWK 5 The orienting reflex and the N2 deflection of the event-related potential (ERP). Adv Psychol (1983) 10:119–41. 10.1016/S0166-4115(08)62036-1

[52] Näätänen R Selective attention and evoked potentials inhumans - A critical review. Biol Psychol (1975) 2(4):237–307. 10.1016/0301-0511(75)90038-1 1156626

[53] Näätänen R Jacobsen T Winkler I Memory-based or afferent processes in mismatch negativity (MMN): a review of the evidence. Psychophysiology (2005) 42(1):25–32. 10.1111/j.1469-8986.2005.00256.x 15720578

[54] Kujala T Tervaniemi M Schroger E The mismatch negativity in cognitive and clinical neuroscience: theoretical and methodological considerations. Biol Psychol (2007) 74(1):1–19. 10.1016/j.biopsycho.2006.06.001 16844278

[55] Ritter W Deacon D Gomes H Javitt DC Vaughan HG The mismatch negativity of event-related potentials as a probe of transient auditory memory: A review. Ear Hear (1995) 16(1):52–67. 10.1097/00003446-199502000-00005 7774769

[56] Cowan N On short and long auditory stores. psychol Bull (1984) 96(2):341–70. 10.1037/0033-2909.96.2.341 6385047

[57] Alho K Tervaniemi M Huotilainen M Lavikainen J Tiitinen H Ilmoniemi RJ Processing of complex sounds in the human auditory cortex as revealed by magnetic brain responses. Psychophysiology (1996) 33(4):369–75. 10.1111/j.1469-8986.1996.tb01061.x 8753936

[58] Alain C Achim A Woods DL Separate memory-related processing for auditory frequency and patterns. Psychophysiology (1999) 36(6):737–44. 10.1017/S0048577299980812 10554587

[59] Ford JM Hillyard SA Event-related potentials (ERPs) to interruptions of a steady rhythm. Psychophysiology (1981) 18(3):322–30. 10.1111/j.1469-8986.1981.tb03043.x 7291450

[60] Saarinen J Paavilainen P Schöger E Tervaniemi M Näätänen R Representation of abstract attributes of auditory stimuli in the human brain: Evidence for primitive intelligence at the sensory level. NeuroReport (1992) 3(12):1149–51. 10.1097/00001756-199212000-00030 1493229

[61] Paavilainen P The mismatch-negativity (MMN) component of the auditory event-related potential to violations of abstract regularities: a review. Int J Psychophysiol (2013) 88(2):109–23. 10.1016/j.ijpsycho.2013.03.015 23542165

[62] Yabe H Tervaniemi M Reinikainen K Näätänen R Temporal window of integration revealed by MMN to sound omission. Neuroreport (1997) 8(8):1971–4. 10.1097/00001756-199705260-00035 9223087

[63] Hughes HC Darcey TM Barkan HI Williamson PD Roberts DW Aslin CH Responses of human auditory association cortex to the omission of an expected acoustic event. NeuroImage (2001) 13(6):1073–89. 10.1006/nimg.2001.0766 11352613

[64] Salisbury DF Finding the missing stimulus mismatch negativity (MMN): emitted MMN to violations of an auditory gestalt. Psychophysiology (2012) 49(4):544–8. 10.1111/j.1469-8986.2011.01336.x PMC3309149 22221004

[65] Horton J Millar A Labelle A Knott VJ MMN responsivity to manipulations of frequency and duration deviants in chronic, clozapine-treated schizophrenia patients. Schizophr Res (2011) 126(1–3):202–11. 10.1016/j.schres.2010.11.028 21194893

[66] Novitski N Tervaniemi M Huotilainen M Näätänen R Frequency discrimination at different frequency levels as indexed by electrophysiological and behavioral measures. Brain Res Cogn Brain Res (2004) 20(1):26–36. 10.1016/j.cogbrainres.2003.12.011 15130586

[67] Näätänen R Alho K Mismatch negativity–the measure for central sound representation accuracy. Audiol Neuro-Otol (1997) 2(5):341–53. 10.1159/000259255 9390839

[68] Tiitinen H May P Reinikainen K Näätänen R Attentive novelty detection in humans is governed by pre-attentive sensory memory. Nature (1994) 372(6501):90–2. 10.1038/372090a0 7969425

[69] Imada T Hari R Loveless N McEvoy L Sams M Determinants of the auditory mismatch response. Electroencephalography Clin Neurophysiol (1993) 87(3):144–53. 10.1016/0013-4694(93)90120-K 7691541

[70] Sabri M Campbell KB Effects of sequential and temporal probability of deviant occurrence on mismatch negativity. Brain Res Cogn Brain Res (2001) 12(1):171–80. 10.1016/S0926-6410(01)00026-X 11489621

[71] Pekkonen E Rinne T Näätänen R Variability and replicability of the mismatch negativity. Electroencephalography Clin Neurophysiol Potentials Section (1995) 96(6):546–54. 10.1016/0013-4694(95)00148-R 7489676

[72] Näätänen R The role of attention in auditory information processing as revealed by event-related potentials and other brain measures of cognitive function. Behav Brain Sci (1990) 13(02):201–33. 10.1017/S0140525X00078407

[73] Paavilainen P Tiitinen H Alho K Näätänen R Mismatch negativity to slight pitch changes outside strong attentional focus. Biol Psychol (1993) 37(1):23–41. 10.1016/0301-0511(93)90025-4 8110919

[74] Alho K Woods DL Algazi A Processing of auditory stimuli during auditory and visual attention as revealed by event-related potentials. Psychophysiology (1994) 31(5):469–79. 10.1111/j.1469-8986.1994.tb01050.x 7972601

[75] Ritter W Paavilainen P Lavikainen J Reinikainen K Alho K Sams M Event-related potentials to repetition and change of auditory stimuli. Electroencephalography Clin Neurophysiol (1992) 83(5):306–21. 10.1016/0013-4694(92)90090-5 1385087

[76] Loewy DH Campbell KB Bastien C The mismatch negativity to frequency deviant stimuli during natural sleep. Electroencephalography Clin Neurophysiol (1996) 98(6):493–501. 10.1016/0013-4694(96)95553-4 8763509

[77] Fischer C Morlet D Bouchet P Luaute J Jourdan C Salord F Mismatch negativity and late auditory evoked potentials in comatose patients. Clin Neurophysiol: Off J Int Fed Clin Neurophysiol (1999) 110(9):1601–10. 10.1016/S1388-2457(99)00131-5 10479027

[78] Nashida T Yabe H Sato Y Hiruma T Sutoh T Shinozaki N Automatic auditory information processing in sleep. Sleep (2000) 23(6):821–8. 10.1093/sleep/23.6.1i 11007449

[79] van Zuijen TL Simoens VL Paavilainen P Näätänen R Tervaniemi M Implicit, intuitive, and explicit knowledge of abstract regularities in a sound sequence: an event-related brain potential study. J Cognit Neurosci (2006) 18(8):1292–303. 10.1162/jocn.2006.18.8.1292 16859415

[80] Alho K Woods DL Algazi A Näätänen R Intermodal selective attention. II. Effects of attentional load on processing of auditory and visual stimuli in central space. Electroencephalography Clin Neurophysiol (1992) 82(5):356–68. 10.1016/0013-4694(92)90005-3 1374704

[81] Näätänen R Tervaniemi M Sussman E Paavilainen P Winkler I “Primitive intelligence” in the auditory cortex. Trends Neurosci (2001) 24(5):283–8. 10.1016/S0166-2236(00)01790-2 11311381

[82] Näätänen R Paavilainen P Titinen H Jiang D Alho K Attention and mismatch negativity. Psychophysiology (1993) 30(5):436–50. 10.1111/j.1469-8986.1993.tb02067.x 8416070

[83] Sussman E Kujala T Halmetoja J Lyytinen H Alku P Näätänen R Automatic and controlled processing of acoustic and phonetic contrasts. Hear Res (2004) 190(1–2):128–40. 10.1016/S0378-5955(04)00016-4 15051135

[84] Woldorff MG Hackley SA Hillyard SA The effects of channel-selective attention on the mismatch negativity wave elicited by deviant tones. Psychophysiology (1991) 28(1):30–42. 10.1111/j.1469-8986.1991.tb03384.x 1886962

[85] Alain C Woods DL Attention modulates auditory pattern memory as indexed by event-related brain potentials. Psychophysiology (1997) 34(5):534–46. 10.1111/j.1469-8986.1997.tb01740.x 9299908

[86] Szymanski MD Yund EW Woods DL Phonemes, intensity and attention: differential effects on the mismatch negativity (MMN). J Acoustical Soc America (1999) 106(6):3492–505. 10.1121/1.428202 10615689

[87] Haenschel C Vernon DJ Dwivedi P Gruzelier JH Baldeweg T Event-related brain potential correlates of human auditory sensory memory-trace formation. J Neurosci (2005) 25(45):10494. 10.1523/JNEUROSCI.1227-05.2005 16280587 PMC6725828

[88] Sussman ES A new view on the MMN and attention debate. J Psychophysiol (2007) 21(3–4):164–75. 10.1027/0269-8803.21.34.164

[89] Näätänen R Alho K Mismatch negativity-a unique measure of sensory processing in audition. Int J Neurosci (1995) 80:317–37. 10.3109/00207459508986107org/10.3109/00207459508986107 7775056

[90] Molholm S Martinez A Ritter W Javitt DC Foxe JJ The Neural Circuitry of Pre-attentive Auditory Change-detection: An fMRI Study of Pitch and Duration Mismatch Negativity generators. Cereb Cortex (2005) 15(5):545–51. 10.1093/cercor/bhh155 15342438

[91] Opitz B Rinne T Mecklinger A von Cramon DY Schröger E Differential Contribution of Frontal and Temporal Cortices to Auditory Change Detection: fMRI and ERP Results. NeuroImage (2002) 15(1):167–74. 10.1006/NIMG.2001.0970 11771985

[92] Knight RT Hillyard SA Woods DL Neville HJ The effects of frontal cortex lesions on event-related potentials during auditory selective attention. Electroencephalography Clin Neurophysiol (1981) 52(6):571–82. 10.1016/0013-4694(81)91431-0 6172256

[93] Chao LL Knight RT Human prefrontal lesions increase distractibility to irrelevant sensory inputs. Neuroreport (1995) 6(12):1605–10. 10.1097/00001756-199508000-00005 8527724

[94] Nobre AC Sebestyen GN Gitelman DR Mesulam MM Frackowiak RS Frith CD Functional localization of the system for visuospatial attention using positron emission tomography. Brain : A J Neurol (1997) 120( Pt 3):515–33. 10.1093/brain/120.3.515 9126062

[95] Rinne T Alho K Ilmoniemi RJ Virtanen J Näätänen R Separate time behaviors of the temporal and frontal mismatch negativity sources. NeuroImage (2000) 12(1):14–9. 10.1006/NIMG.2000.0591 10875898

[96] Tervaniemi M Hugdahl K Lateralization of auditory-cortex functions. Brain Res Rev (2003) 43:231–46. 10.1016/j.brainresrev.2003.08.004 14629926

[97] Knight RT Contribution of human hippocampal region to novelty detection. Nature (1996) 383(6597):256–9. 10.1038/383256a0 8805701

[98] Soltani M Knight RT Neural origins of the P300. Crit Rev Neurobiol (2000) 14(3–4):199–224. 10.1615/CritRevNeurobiol.v14.i3-4.20 12645958

[99] Friedman D Cycowicz YM Gaeta H The novelty P3: an event-related brain potential (ERP) sign of the brain's evaluation of novelty. Neurosci Biobehav Rev (2001) 25(4):355–73. 10.1016/S0149-7634(01)00019-7 11445140

[100] Linden DEJ The P300: Where in the Brain Is It Produced and What Does It Tell Us? Neuroscientist (2005) 11(6):563–76. 10.1177/1073858405280524 16282597

[101] Escera C Alho K Schröger E Winkler I Involuntary attention and distractibility as evaluated with event-related brain potentials. Audiol Neuro-Otol (2000) 5(3–4):151–66. 10.1159/000013877 10859410

[102] Näätänen R Winkler I The concept of auditory stimulus representation in cognitive neuroscience. psychol Bull (1999) 125(6):826–59. 10.1037/0033-2909.125.6.826 10589304

[103] Squires KC Hillyard SA Lindsay PH Vertex potentials evoked during auditory signal detection: Relation to decision criteria. Percept Psychophysics (1973) 14(2):265–72. 10.3758/BF03212388

[104] Johnson R Donchin E On how P300 amplitude varies with the utility of the eliciting stimuli. Electroencephalography Clin Neurophysiol (1978) 44(4):424–37. 10.1016/0013-4694(78)90027-5 76551

[105] Fitzgerald PG Picton TW The Effects of Probability and Discriminability on the Evoked Potentials to Unpredictable Stimuli. Ann New York Acad Sci (1984) 425(1 Brain and Inf):199–203. 10.1111/j.1749-6632.1984.tb23533.x 6588830

[106] Squires NK Squires KC Hillyard SA Two varieties of long-latency positive waves evoked by unpredictable auditory stimuli in man. Electroencephalography Clin Neurophysiol (1975) 38(4):387–401. 10.1016/0013-4694(75)90263-1 46819

[107] Courchesne E Hillyard SA Courchesne RY P3 Waves to the Discrimination of Targets in Homogeneous and Heterogeneous Stimulus Sequences. Psychophysiology (1977) 14(6):590–7. 10.1111/j.1469-8986.1977.tb01206.x 928611

[108] Duncan-Johnson CC Donchin E On Quantifying Surprise: The Variation of Event-Related Potentials With Subjective Probability. Psychophysiology (1977) 14(5):456–67. 10.1111/j.1469-8986.1977.tb01312.x 905483

[109] Squires KC Wickens C Squires NK Donchin E The effect of stimulus sequence on the waveform of the cortical event-related potential. Sci (New York NY) (1976) 193(4258):1142–6. 10.1126/science.959831 959831

[110] Kok A On the utility of P3 amplitude as a measure of processing capacity. Psychophysiology (2001) 38(3):S0048577201990559. 10.1017/S0048577201990559 11352145

[111] Rosburg T Weigl M Thiel R Mager R The event-related potential component P3a is diminished by identical deviance repetition, but not by non-identical repetitions. Exp Brain Res (2018) 236(5):1519–30. 10.1007/s00221-018-5237-z 29564505

[112] Winkler I Tervaniemi M Näätänen R Two separate codes for missing-fundamental pitch in the human auditory cortex. J Acoustical Soc America (1998) 102(2):1072. 10.1121/1.419860 9265755

[113] Sussman E Winkler I Schröger E Top-down control over involuntary attention switching in the auditory modality. Psychonomic Bull Rev (2003) 10(3):630–7. 10.3758/BF03196525 14620357

[114] Rinne T Särkkä A Degerman A Schröger E Alho K Two separate mechanisms underlie auditory change detection and involuntary control of attention. Brain Res (2006) 1077(1):135–43. 10.1016/J.BRAINRES.2006.01.043 16487946

[115] Gaeta H Friedman D Ritter W Cheng J An event-related potential evaluation of involuntary attentional shifts in young and older adults. Psychol Aging (2001) 16(1):55–68. 10.1037/0882-7974.16.1.55 11302368

[116] Horváth J Winkler I Bendixen A Do N1/MMN, P3a, and RON form a strongly coupled chain reflecting the three stages of auditory distraction? Biol Psychol (2008) 79(2):139–47. 10.1016/j.biopsycho.2008.04.001 18468765

[117] Sussman E Winkler I Wang W MMN and attention: Competition for deviance detection. Psychophysiology (2003) 40(3):430–5. 10.1111/1469- 12946116

[118] Näätänen R Michie PT Early selective-attention effects on the evoked potential: A critical review and reinterpretation. Biol Psychol (1979) 8(2):81–136. 10.1016/0301-0511(79)90053-X 465623

[119] Doeller CF Opitz B Mecklinger A Krick C Reith W Schröger E Prefrontal cortex involvement in preattentive auditory deviance detection. NeuroImage (2003) 20(2):1270–82. 10.1016/S1053-8119(03)00389-6 14568496

[120] Garrido MI Friston KJ Kiebel SJ Stephan KE Baldeweg T Kilner JM The functional anatomy of the MMN: a DCM study of the roving paradigm. NeuroImage (2008) 42(2):936–44. 10.1016/j.neuroimage.2008.05.018 PMC2640481 18602841

[121] Garrido MI Kilner JM Kiebel SJ Friston KJ Dynamic Causal Modeling of the Response to Frequency Deviants. J Neurophysiol (2009) 101(5):2620–31. 10.1152/jn.90291.2008 PMC2681422 19261714

[122] Garrido MI Kilner JM Kiebel SJ Stephan KE Baldeweg T Friston KJ Repetition suppression and plasticity in the human brain. NeuroImage (2009) 48(1):269–79. 10.1016/j.neuroimage.2009.06.034 PMC2821573 19540921

[123] Hari R Hämäläinen M Ilmoniemi R Kaukoranta E Reinikainen K Salminen J Responses of the primary auditory cortex to pitch changes in a sequence of tone pips: neuromagnetic recordings in man. Neurosci Lett (1984) 50(1–3):127–32. 10.1016/0304-3940(84)90474-9 6493619

[124] Paavilainen P Mikkonen M Kilpeläinen M Lehtinen R Saarela M Tapola L Evidence for the different additivity of the temporal and frontal generators of mismatch negativity: a human auditory event-related potential study. Neurosci Lett (2003) 349(2):79–82. 10.1016/s0304-3940(03)00787-0 12946557

[125] Shalgi S Deouell LY Direct evidence for differential roles of temporal and frontal components of auditory change detection. Neuropsychologia (2007) 45(8):1878–88. 10.1016/j.neuropsychologia.2006.11.023 17239410

[126] Dittmann-Balçar A Jüptner M Jentzen W Schall U Dorsolateral prefrontal cortex activation during automatic auditory duration-mismatch processing in humans: a positron emission tomography study. Neurosci Lett (2001) 308(2):119–22. 10.1016/S0304-3940(01)01995-4 11457574

[127] Müller BW Jüptner M Jentzen W Müller SP Cortical activation to auditory mismatch elicited by frequency deviant and complex novel sounds: a PET study. NeuroImage (2002) 17(1):231–9. 10.1006/nimg.2002.1176 12482080

[128] Levänen S Ahonen A Hari R McEvoy L Sams M Deviant auditory stimuli activate human left and right auditory cortex differently. Cereb Cortex (New York NY : 1991) (1996) 6(2):288–96. 10.1093/cercor/6.2.288 8670657

[129] Celsis P Boulanouar K Doyon B Ranjeva JP Berry I Nespoulous JL Differential fMRI Responses in the Left Posterior Superior Temporal Gyrus and Left Supramarginal Gyrus to Habituation and Change Detection in Syllables and Tones. NeuroImage (1999) 9(1):135–44. 10.1006/nimg.1998.0389 9918735

[130] Schall U Johnston P Todd J Ward PB Michie PT Functional neuroanatomy of auditory mismatch processing: an event-related fMRI study of duration-deviant oddballs. NeuroImage (2003) 20(2):729–36. 10.1016/S1053-8119(03)00398-7 14568447

[131] Tse C-Y Tien K-R Penney TB Event-related optical imaging reveals the temporal dynamics of right temporal and frontal cortex activation in pre-attentive change detection. NeuroImage (2006) 29(1):314–20. 10.1016/j.neuroimage.2005.07.013 16095922

[132] Alho K Woods DL Algazi A Knight RT Näätänen R Lesions of frontal cortex diminish the auditory mismatch negativity. Electroencephalography Clin Neurophysiol (1994) 91(5):353–62. 10.1016/0013-4694(94)00173-1 7525232

[133] Chen JC Hämmerer D Strigaro G Liou LM Tsai CH Rothwell JC Domain-specific suppression of auditory mismatch negativity with transcranial direct current stimulation. Clin Neurophysiol (2014) 125(3):585–92. 10.1016/J.CLINPH.2013.08.007 24051072

[134] Wolff C Schroger E Activation of the auditory pre-attentive change detection system by tone repetitions with fast stimulation rate. Cogn Brain Res (2001) 10:323–7. 10.1016/S0926-6410(00)00043-4 11167055

[135] Schönwiesner M Novitski N Pakarinen S Carlson S Tervaniemi M Näätänen R Heschl's Gyrus, Posterior Superior Temporal Gyrus, and Mid-Ventrolateral Prefrontal Cortex have different roles in the detection of acoustic changes. J Neurophysiol (2007) 97(3):2075–82. 10.1152/jn.01083.2006 17182905

[136] Heilman KM Van Den Abell T Right hemisphere dominance for attention: the mechanism underlying hemispheric asymmetries of inattention (neglect). Neurology (1980) 30(3):327–30. 10.1212/WNL.30.3.327 7189037

[137] Posner MI Orienting of attention. Q J Exp Psychol (1980) 32(1):3–25. 10.1080/00335558008248231 7367577

[138] Mesulam M-M A cortical network for directed attention and unilateral neglect. Ann Neurol (1981) 10(4):309–25. 10.1002/ana.410100402 7032417

[139] Kastner S Ungerleider LG Mechanisms of Visual Attention in the Human Cortex. Annu Rev Neurosci (2000) 23(1):315–41. 10.1146/annurev.neuro.23.1.315 10845067

[140] Deouell LY The frontal generator of the mismatch negativity revisited. J Psychophysiol (2007) 21(3-4):188–203. 10.1027/0269-8803.21.34.188

[141] Halgren E Sherfey J Irimia A Dale AM Marinkovic K Sequential temporo-fronto-temporal activation during monitoring of the auditory environment for temporal patterns. Hum Brain Mapp (2011) 32(8):1260–76. 10.1002/hbm.21106 PMC2970649 20665718

[142] Schröger E Bendixen A Trujillo-Barreto NJ Roeber U Processing of Abstract Rule Violations in Audition. PloS One (2007) 2(11):e1131. 10.1371/journal.pone.0001131 17987118 PMC2043487

[143] Wacongne C Labyt E van Wassenhove V Bekinschtein T Naccache L Dehaene S Evidence for a hierarchy of predictions and prediction errors in human cortex. Proc Natl Acad Sci (2011) 108(51):20754–9. 10.1073/pnas.1117807108 PMC3251061 22147913

[144] May P Tiitinen H Mismatch negativity (MMN), the deviance-elicited auditory deflection, explained. Psychophysiology (2010) 47(1):66–122. 10.1111/j.1469-8986.2009.00856.x 19686538

[145] Fishman YI The Mechanisms and Meaning of the Mismatch Negativity. Brain Topography (2014) 27(4):500–26. 10.1007/s10548-013-0337-3 24276221

[146] Sams M Hari R Rif J Knuutila J The Human Auditory Sensory Memory Trace Persists about 10 sec: Neuromagnetic Evidence. J Cognit Neurosci (1993) 5(3):363–70. 10.1162/jocn.1993.5.3.363 23972223

[147] Winkler I Schröger E Cowan N The Role of Large-Scale Memory Organization in the Mismatch Negativity Event-Related Brain Potential. J Cogn Neurosci (2001) 13(1):59–71. 10.1162/089892901564171 11224909

[148] Glucksberg S Cowen GN Memory for nonattended auditory material. Cogn Psychol (1970) 1(2):149–56. 10.1016/0010-0285(70)90010-1

[149] Netser S Zahar Y Gutfreund Y Stimulus-specific adaptation: can it be a neural correlate of behavioral habituation? J Neurosci : Off J Soc Neurosci (2011) 31(49):17811–20. 10.1523/JNEUROSCI.4790-11.2011 PMC6634140 22159097

[150] Ayala YA Malmierca MS Stimulus-specific adaptation and deviance detection in the inferior colliculus. Front Neural Circuits (2013) 6:89. 10.3389/fncir.2012.00089 23335883 PMC3547232

[151] Hasson U Yang E Vallines I Heeger DJ Rubin N A hierarchy of temporal receptive windows in human cortex. J Neurosci : Off J Soc Neurosci (2008) 28(10):2539–50. 10.1523/JNEUROSCI.5487-07.2008 PMC2556707 18322098

[152] Kiebel SJ Daunizeau J Friston KJ A hierarchy of time-scales and the brain. PloS Comput Biol (2008) 4(11):e1000209. 10.1371/journal.pcbi.1000209 19008936 PMC2568860

[153] Winkler I Cowan N Csépe V Czigler I Näätänen R Interactions between transient and long-term auditory memory as reflected by the mismatch negativity. J Cogn Neurosci (1996) 8(5):403–15. 10.1162/jocn.1996.8.5.403 23961944

[154] Winkler I Czigler I Mismatch negativity: Deviance detection or the maintenance of the “standard”. NeuroReport (1998) 9(17):3809–13. 10.1097/00001756-199812010-00008 9875709

[155] Kok P Failing MF de Lange FP Prior expectations evoke stimulus templates in the primary visual cortex. J Cogn Neurosci (2014) 26(7):1546–54. 10.1162/jocn_a_00562 24392894

[156] Tervaniemi M Maury S Näätänen R Neural representations of abstract stimulus features in the human brain as reflected by the mismatch negativity. Neuroreport (1994) 5(7):844–6. 10.1097/00001756-199403000-0002 8018861

[157] Ulanovsky N Las L Nelken I Processing of low-probability sounds by cortical neurons. Nat Neurosci (2003) 6(4):391–8. 10.1038/nn1032 12652303

[158] May P Tiitinen H Ilmoniemi RJ Nyman G Taylor JG Näätänen R Frequency change detection in human auditory cortex. J Comput Neurosci (1999) 6(2):99–120. 10.1023/A:1008896417606 10333158

[159] Ulanovsky N Las L Farkas D Nelken I Multiple time scales of adaptation in auditory cortex neurons. J Neurosci (2004) 24(46):10440–53. 10.1523/jneurosci.1905-04.2004 PMC6730303 15548659

[160] Costa-Faidella J Grimm S Slabu L Díaz-Santaella F Escera C Multiple time scales of adaptation in the auditory system as revealed by human evoked potentials. Psychophysiology (2011) 48(6):774–83. 10.1111/j.1469-8986.2010.01144.x 20946129

[161] Jacobsen T Schröger E Is there pre-attentive memory-based comparison of pitch? Psychophysiology (2001) 38(4):723–7. 10.1111/1469-8986.3840723 11446587

[162] Maess B Jacobsen T Schröger E Friederici AD Localizing pre-attentive auditory memory-based comparison: Magnetic mismatch negativity to pitch change. NeuroImage (2007) 37(2):561–71. 10.1016/j.neuroimage.2007.05.040 17596966

[163] Edwards E Soltani M Deouell LY Berger MS Knight RT High gamma activity in response to deviant auditory stimuli recorded directly from human cortex. J Neurophysiol (2005) 94(6):4269–80. 10.1152/jn.00324.2005 16093343

[164] Fishman YI Steinschneider M Searching for the mismatch negativity in primary auditory cortex of the awake monkey: deviance detection or stimulus specific adaptation? J Neurosci : Off J Soc Neurosci (2012) 32(45):15747–58. 10.1523/JNEUROSCI.2835-12.2012 PMC3641775 23136414

[165] Taaseh N Yaron A Nelken I Stimulus-specific adaptation and deviance detection in the rat auditory cortex. PloS One (2011) 6(8):e23369. 10.1371/journal.pone.0023369 21853120 PMC3154435

[166] Harms L Fulham WR Todd J Budd TW Hunter M Meehan C Mismatch negativity (MMN) in freely-moving rats with several experimental controls. PloS One (2014) 9(10):e110892. 10.1371/journal.pone.0110892 25333698 PMC4205004

[167] Althen H Grimm S Escera C Fast detection of unexpected sound intensity decrements as revealed by human evoked potentials. PloS One (2011) 6(12):e28522. 10.1371/journal.pone.0028522 22163029 PMC3232232

[168] Lozano-Soldevilla D Marco-Pallarés J Fuentemilla L Grau C Common N1 and mismatch negativity neural evoked components are revealed by independent component model-based clustering analysis. Psychophysiology (2012) 49(11):1622–31. 10.1111/j.1469-8986.2012.01458.x 22971105

[169] Jääskeläinen IP Ahveninen J Bonmassar G Dale AM Ilmoniemi RJ Levänen S Human posterior auditory cortex gates novel sounds to consciousness. Proc Natl Acad Sci United States America (2004) 101(17):6809–14. 10.1073/pnas.0303760101 PMC404127 15096618

[170] Horváth J Winkler I How the human auditory system treats repetition amongst change. Neurosci Lett (2004) 368(2):157–61. 10.1016/j.neulet.2004.07.004 15351440

[171] Wacongne C Changeux JP Dehaene S A neuronal model of predictive coding accounting for the mismatch negativity. J Neurosci (2012) 32(11):3665–78. 10.1523/JNEUROSCI.5003-11.2012 PMC6703454 22423089

[172] Macdonald M Campbell K Effects of a violation of an expected increase or decrease in intensity on detection of change within an auditory pattern. Brain Cogn (2011) 77(3):438–45. 10.1016/j.bandc.2011.08.014 21925782

[173] Sussman ES Gumenyuk V Organization of sequential sounds in auditory memory. NeuroReport (2005) 16(13):1519–23. 10.1097/01.wnr.0000177002.35193.4c 16110282

[174] Sussman E Ritter W Vaughan HG Predictability of stimulus deviance and the mismatch negativity. Neuroreport (1998) 9(18):4167–70. 10.1097/00001756-199812210-00031 9926868

[175] Jacobsen T Schröger E Measuring duration mismatch negativity. Clin Neurophysiol (2003) 114(6):1133–43. 10.1016/s1388-2457(03)00043-9 12804682

[176] Parras GG Nieto-Diego J Carbajal GV Valdés-Baizabal C Escera C Malmierca MS Neurons along the auditory pathway exhibit a hierarchical organization of prediction error. Nat Commun (2017) 8:2148. 10.1038/s41467-017-02038-6 29247159 PMC5732270

[177] Kurkela JLO Lipponen A Kyläheiko I Astikainen P Electrophysiological evidence of memory-based detection of auditory regularity violations in anesthetized mice. Sci Rep 8 (2018) 8:3027. 10.1038/s41598-018-21411-z PMC5813195 29445171

[178] Carbajal GV Malmierca MS The neuronal basis of predictive coding along the auditory pathway: From the subcortical roots to cortical deviance detection. Trends Hear (2018) 22(2331216518784822). 10.1177/2331216518784822 PMC6053868 30022729

[179] Mittag M Takegata R Winkler I Transitional Probabilities Are Prioritized over Stimulus/Pattern Probabilities in Auditory Deviance Detection: Memory Basis for Predictive Sound Processing. J Neurosci : Off J Soc Neurosci (2016) 36(37):9572–9. 10.1523/JNEUROSCI.1041-16.2016 PMC6601944 27629709

[180] Friston K Learning and inference in the brain. Neural Netw (2003) 16(9):1325–52. 10.1016/j.neunet.2003.06.005 14622888

[181] Mumford D On the computational architecture of the neocortex. Biol Cybernet (1992) 66(3):241–51. 10.1007/BF00198477 1540675

[182] Gregory RL Perceptions as hypotheses. Philos Trans R Soc London Ser B Biol Sci (1980) 290(1038):181–97. 10.1098/rstb.1980.0090 6106237

[183] Garrido MI Kilner JM Kiebel SJ Stephan KE Friston KJ Dynamic causal modelling of evoked potentials: a reproducibility study. NeuroImage (2007) 36(3):571–80. 10.1016/j.neuroimage.2007.03.014 PMC2640482 17478106

[184] Friston K The free-energy principle: a unified brain theory? Nat Rev Neurosci (2010) 11(2):127–38. 10.1038/nrn2787 20068583

[185] Friston K Hierarchical models in the brain. PloS Comput Biol (2008) 4(11):e1000211. 10.1371/journal.pcbi.1000211 18989391 PMC2570625

[186] Kersten D Mamassian P Yuille A Object Perception as Bayesian Inference. Annu Rev Psychol (2004) 55(1):271–304. 10.1146/annurev.psych.55.090902.142005 14744217

[187] Friston K Kiebel S Cortical circuits for perceptual inference. Neural Networks (2009) 22(8):1093–104. 10.1016/j.neunet.2009.07.023 PMC2796185 19635656

[188] Feldman H Friston KJ Attention, Uncertainty, and Free-Energy. Front Hum Neurosci (2010) 4:215. 10.3389/fnhum.2010.00215 21160551 PMC3001758

[189] Kalman RE A New Approach to Linear Filtering and Prediction Problems. J Basic Eng (1960) 82(1):35. 10.1115/1.3662552

[190] Gurtubay IG Alegre M Valencia M Artieda J Cortical gamma activity during auditory tone omission provides evidence for the involvement of oscillatory activity in top-down processing. Exp Brain Res (2006) 175(3):463–70. 10.1007/s00221-006-0561-0 16763832

[191] Wyart V Tallon-Baudry C How Ongoing Fluctuations in Human Visual Cortex Predict Perceptual Awareness: Baseline Shift versus Decision Bias. J Neurosci (2009) 29(27):8715–25. 10.1523/JNEUROSCI.0962-09.2009 PMC6664890 19587278

[192] Desimone R Neural mechanisms for visual memory and their role in attention. Proc Natl Acad Sci United States America (1996) 93(24):13494–9. 10.1073/pnas.93.24.13494 PMC33636 8942962

[193] Baldeweg T Repetition effects to sounds: evidence for predictive coding in the auditory system. Trends Cogn Sci (2006) 10(3):93–4. 10.1016/j.tics.2006.01.010 16460994

[194] Todorovic A de Lange FP Repetition suppression and expectation suppression are dissociable in time in early auditory evoked fields. J Neurosci : Off J Soc Neurosci (2012) 32(39):13389–95. 10.1523/JNEUROSCI.2227-12.2012 PMC6621367 23015429

[195] Liégeois-Chauvel C Musolino A Badier JM Marquis P Chauvel P Evoked potentials recorded from the auditory cortex in man: evaluation and topography of the middle latency components. Electroencephalography Clin Neurophysiol (1994) 92(3):204–14. 10.1016/0168-5597(94)90064-7 7514990

[196] Knight RT Decreased response to novel stimuli after prefrontal lesions in man. Electroencephalography Clin Neurophysiol Potentials Section (1984) 59(1):9–20. 10.1016/0168-5597(84)90016-9 6198170

[197] Yamaguchi S Knight RT Gating of somatosensory input by human prefrontal cortex. Brain Res (1990) 521(1–2):281–8. 10.1016/0006-8993(90)91553-S 2207666

[198] Javitt DC Steinschneider M Schroeder CE Arezzo JC Role of cortical N-methyl-D-aspartate receptors in auditory sensory memory and mismatch negativity generation: implications for schizophrenia. Proc Natl Acad Sci United States America (1996) 93(21):11962–7. 10.1073/pnas.93.21.11962 PMC38166 8876245

[199] Umbricht D Schmid L Koller R Vollenweider FX Hell D Javitt DC Ketamine-induced deficits in auditory and visual context-dependent processing in healthy volunteers: implications for models of cognitive deficits in schizophrenia. Arch Gen Psychiatry (2000) 57(12):1139–47. 10.1001/archpsyc.57.12.1139 11115327

[200] Näätänen R Sussman ES Salisbury D Shafer VL Mismatch negativity (MMN) as an index of cognitive dysfunction. Brain Topogr (2014) 27(4):451–66. 10.1007/s10548-014-0374-6 PMC4096841 24838819

[201] Bendixen A Roeber U Schröger E Regularity extraction and application in dynamic auditory stimulus sequences. J Cogn Neurosci (2007) 19(10):1664–77. 10.1162/jocn.2007.19.10.1664 18271740

[202] O'Shea RP Refractoriness about adaptation. Front Hum Neurosci (2015). 9(38). 10.3389/fnhum.2015.00038 PMC4316709 25698961

[203] Stefanics G Kremláček J Czigler I Mismatch negativity and neural adaptation: Two sides of the same coin. Response: Commentary: Visual mismatch negativity: a predictive coding view. Front Hum Neurosci (2016) 10:13. 10.3389/fnhum.2016.00013 26858625 PMC4732183

[204] Nelken I Ulanovsky N Mismatch Negativity and Stimulus-Specific Adaptation in Animal Models. J Psychophysiol (2007) 21(3–4):214–23. 10.1027/0269-8803.21.34.214

[205] Escera C Malmierca MS The auditory novelty system: an attempt to integrate human and animal research. Psychophysiology (2014) 51(2):111–23. 10.1111/psyp.12156 24423134

[206] Nelken I Stimulus-specific adaptation and deviance detection in the auditory system: experiments and models. Biol Cybernet (2014) 108(5):655–63. 10.1007/s00422-014-0585-7 24477619

[207] Frost JD Haasnoot K McDonnell K Winkler I Todd J The cognitive resource and foreknowledge dependence of auditory perceptual inference. Neuropsychologia (2018) 117:379–88. 10.1016/J.NEUROPSYCHOLOGIA.2018.07.005 29981292

[208] Fitzgerald K Provost A Todd J First-impression bias effects on mismatch negativity to auditory spatial deviants. Psychophysiology (2018) 55(4):e13013. 10.1111/psyp.13013 28972671

[209] Todd J Heathcote A Mullens D Whitson LR Provost A Winkler I What controls gain in gain control? Mismatch negativity (MMN), priors and system biases. Brain Topography (2014) 27(4):578–89. 10.1007/s10548-013-0344-4 24343248

[210] Todd J Frost J Yeark M Paton B Context is everything: How context shapes modulation of responses to unattended sound. Hear Res (2020). 10.1016/j.heares.2020.107975 32370880

[211] Brown HR Friston KJ The functional anatomy of attention: a DCM study. Front Hum Neurosci (2013) 7:784. 10.3389/fnhum.2013.00784 24348359 PMC3845206

[212] Mullens D Woodley J Whitson L Provost A Heathcote A Winkler I Altering the primacy bias–how does a prior task affect mismatch negativity? Psychophysiology (2014) 51(5):437–45. 10.1111/psyp.12190 24611446

[213] Todd J Petherbridge A Speirs B Provost A Paton B ‘Time as context: The influence of hierarchical patterning on sensoryinference'. Schizophr Res (2018) 191:123–31. 10.1016/j.schres.2017.03.033 28343741

[214] Sussman E Sheridan K Kreuzer J Winkler I Representation of the standard: Stimulus context effects on the process generating the mismatch negativity component of event-related brain potentials. Psychophysiology (2003) 40(3):465–71. 10.1111/1469-8986.00048 12946119

[215] Fitzgerald K Todd J Hierarchical timescales of statistical learning revealed by mismatch negativity to auditory pattern deviations. Neuropsychologia (2019) 120:25–34. 10.1016/j.neuropsychologia.2018.09.015 30268879

[216] Winkler I Korzyukov O Gumenyuk V Cowan N Linkenkaer-Hansen K Ilmoniemi J Temporary and longer term retention of acoustic information. Psychophysiology (2002) 39(4):530–4. 10.1017/S0048577201393186 12212645

[217] Rescorla RA Wagner AR A theory of Pavlovian conditioning: Variations in the effectiveness of reinforcement and nonreinforcement. Classical Conditioning II Curr Res Theory (1972) 21(6):64–99. 10.1101/gr.110528.110

[218] Mathys C Daunizeau J Friston KJ Stephan KE A Bayesian foundation for individual learning under uncertainty. Front Hum Neurosci (2011) 5:39. 10.3389/fnhum.2011.00039 21629826 PMC3096853

[219] Mathys CD Lomakina EI Daunizeau J Iglesias S Brodersen KH Friston KJ Uncertainty in perception and the Hierarchical Gaussian Filter. Front Hum Neurosci (2014) 8:825. 10.3389/fnhum.2014.00825 25477800 PMC4237059

[220] Horváth J Czigler I Sussman E Winkler I Simultaneously active pre-attentive representations of local and global rules for sound sequences in the human brain. Cogn Brain Res (2001) 12(1):131–44. 10.1016/S0926-6410(01)00038-6 11489616

[221] Atienza M Cantero JL Dominguez-Marin E Mismatch negativity (MMN): an objective measure of sensory memory and long-lasting memories during sleep. Int J Psychophysiol (2002) 46:215–25. 10.1016/S0167-8760(02)00113-7 12445949

[222] Light GA Swerdlow NR Future clinical uses of neurophysiological biomarkers to predict and monitor treatment response for schizophrenia. Ann New York Acad Sci (2015) 1344(1):105–19. 10.1111/nyas.12730 PMC4412775 25752648

[223] Tremblay K Kraus N McGee T The time course of auditory perceptual learning: neurophysiological changes during speech-sound training. Neuroreport (1998) 9(16):3557–60. 10.1097/00001756-199811160-00003 9858359

[224] Koelsch S Schröger E Tervaniemi M Superior pre-attentive auditory processing in musicians. Neuroreport (1999) 10(6):1309–13. 10.1097/00001756-199904260-00029 10363945

[225] Cooper RJ Todd J McGill K Michie PT Auditory sensory memory and the aging brain: A mismatch negativity study. Neurobiol Aging (2006) 27(5):752–62. 10.1016/j.neurobiolaging.2005.03.012 15908049

[226] Ruzzoli M Pirulli C Brignani D Maioli C Miniussi C Sensory memory during physiological aging indexed by mismatch negativity (MMN). Neurobiol Aging (2012) 33(3):625.e21–625.e30. 10.1016/j.neurobiolaging.2011.03.021 21530002

[227] Todd J Michie PT Schall U Ward PB Catts SV,J Mismatch negativity (MMN) reduction in schizophrenia: Impaired prediction-error generation, estimation or salience? Int J Psychophysiol (2012) 83:222–31. 10.1016/j.ijpsycho.2011.10.003 22020271

[228] Schall U Is it time to move mismatch negativity into the clinic? Biol Psychol (2016) 116:41–6. 10.1016/j.biopsycho.2015.09.001 26342995

[229] Cheour M Leppänen PH Kraus N Mismatch negativity (MMN) as a tool for investigating auditory discrimination and sensory memory in infants and children. Clin Neurophysiol: Off J Int Fed Clin Neurophysiol (2000) 111(1):4–16. 10.1016/S1388-2457(99)00191-1 10656505

[230] Braff D Light G Preattentional and attentional cognitive deficits as targets for treating schizophrenia. Psychopharmacology (2004) 174(1):75–85. 10.1007/s00213-004-1848-0 15118804

[231] Erickson MA Ruffle A Gold JM A meta-analysis of mismatch negativity in schizophrenia: From clinical risk to disease specificity and progression. Biol Psychiatry (2016) 79(12):980–7. 10.1016/j.biopsych.2015.08.025 PMC4775447 26444073

[232] Coffman BA Haigh SM Murphy TK Salisbury DF Impairment in mismatch negativity but not repetition suppression in schizophrenia. Brain Topography (2017) 30(4):521–30. 10.1007/s10548-017-0571-1 PMC5533081 28516227

[233] McCleery A Mathalon DH Wynn JK Roach BJ Hellemann GS Marder SR Parsing components of auditory predictive coding in schizohprenia using a roving standard mismatch negativity paradigm. psychol Med (2019) 49(7):1195–206. 10.1017/S0033291718004087 PMC6499668 30642411

[234] Rentzsch J Shen C Jockers-Scherübl MC Gallinat J Neuhaus AH Auditory mismatch negativity and repetition suppression deficits in schizophrenia explained by irregular computation of prediction error. PloS One (2015) 10(5):e0126775. 10.1371/journal.pone.0126775 25955846 PMC4425493

[235] Dima D Frangou S Burge L Braeutigam S James AC Abnormal intrinsic and extrinsic connectivity within the magnetic mismatch negativity brain network in schizophrenia: A preliminary study. Schizophr Res (2012) 135(1–3):23–7. 10.1016/j.schres.2011.12.024 22264684

[236] Randeniya R Oestreich LKL Garrido MI Sensory prediction erros in the continuum of psychosis. Schizophr Res (2018) 191:109–22. 10.1016/j.schres.2017.04.019 28457774

[237] Ranlund S Adams RA Diez A Constante M Dutt A Hall MH Impaired prefrontal synaptic gain in people with psychosis andtheir relatives during the mismatch negativity. Hum Brain Mapp (2016) 37(1):351–65. 10.1002/hbm.23035 PMC4843949 26503033

[238] Friston K Brown HR Siemerkus J Stephan KE The dysconnection hypothesis (2016). Schizophr Res (2016) 176(2–3):83–94. 10.1016/j.schres.2016.07.014 27450778 PMC5147460

